# Functional Polymorphisms in DNA Repair Genes Are Associated with Sporadic Colorectal Cancer Susceptibility and Clinical Outcome

**DOI:** 10.3390/ijms20010097

**Published:** 2018-12-27

**Authors:** Katerina Jiraskova, David J. Hughes, Stefanie Brezina, Tanja Gumpenberger, Veronika Veskrnova, Tomas Buchler, Michaela Schneiderova, Miroslav Levy, Vaclav Liska, Sona Vodenkova, Cornelia Di Gaetano, Alessio Naccarati, Barbara Pardini, Veronika Vymetalkova, Andrea Gsur, Pavel Vodicka

**Affiliations:** 1 Institute of Biology and Medical Genetics, First Faculty of Medicine, Charles University, Albertov 4, 128 00 Prague, Czech Republic; sona.vodenkova@iem.cas.cz (S.V.); veronika.vymetalkova@iem.cas.cz (V.V.); 2 Department of Molecular Biology of Cancer, Institute of Experimental Medicine of the Czech Academy of Sciences, Videnska 1083, 142 00 Prague, Czech Republic; alessio.naccarati@iigm.it; 3 Cancer Biology and Therapeutics Group, UCD Conway Institute, University College Dublin, Dublin 4, Ireland; david.hughes@ucd.ie; 4 Institute of Cancer Research, Department of Medicine I, Medical University of Vienna, Borschkegasse 8a, A-1090 Vienna, Austria; stefanie.brezina@meduniwien.ac.at (S.B.); tanja.gumpenberger@meduniwien.ac.at (T.G.); andrea.gsur@meduniwien.ac.at (A.G.); 5 Department of Oncology, First Faculty of Medicine, Charles University and Thomayer Hospital, Videnska 800, 140 59 Prague, Czech Republic; veskrnova.ver@gmail.com (V.V.); buchlert@yahoo.com (T.B.);; 6 Department of Surgery, General University Hospital in Prague, U Nemocnice 499/2, 128 08 Prague, Czech Republic; schneider77@seznam.cz; 7 Department of Surgery, First Faculty of Medicine, Charles University and Thomayer Hospital, Thomayerova 815/5, 140 00 Prague, Czech Republic; miroslav.levy@lf1.cuni.cz; 8 Biomedical Centre, Faculty of Medicine in Pilsen, Charles University in Prague, 323 00 Pilsen, Czech Republic; vena.liska@skaut.cz; 9 Department of Surgery, Medical School in Pilsen, Charles University, Alej svobody 80, 304 600 Pilsen, Czech Republic; 10 Department of Medical Genetics, Third Faculty of Medicine, Charles University, Ruska 2411/87, 100 00 Prague, Czech Republic; 11 Molecular and Genetic Epidemiology; Genomic Variation in Human Populations and Complex Diseases, IIGM Italian Institute for Genomic Medicine, Via Nizza 52, 10126 Turin, Italy; cdigaeta@unito.it (C.D.G.); barbara.pardini@iigm.it (B.P.); 12 Department of Medical Sciences, University of Turin, Corso Dogliotti 14, 10126 Turin, Italy

**Keywords:** DNA repair genes, functional single nucleotide polymorphism, colorectal cancer susceptibility, survival analysis

## Abstract

DNA repair processes are involved in both the onset and treatment efficacy of colorectal cancer (CRC). A change of a single nucleotide causing an amino acid substitution in the corresponding protein may alter the efficiency of DNA repair, thus modifying the CRC susceptibility and clinical outcome. We performed a candidate gene approach in order to analyze the association of non-synonymous single nucleotide polymorphisms (nsSNPs) in the genes covering the main DNA repair pathways with CRC risk and clinical outcome modifications. Our candidate polymorphisms were selected according to the foremost genomic and functional prediction databases. Sixteen nsSNPs in 12 DNA repair genes were evaluated in cohorts from the Czech Republic and Austria. Apart from the tumor-node-metastasis (TNM) stage, which occurred as the main prognostic factor in all of the performed analyses, we observed several significant associations of different nsSNPs with survival and clinical outcomes in both cohorts. However, only some of the genes (*REV3L*, *POLQ,* and *NEIL3*) were prominently defined as prediction factors in the classification and regression tree analysis; therefore, the study suggests their association for patient survival. In summary, we provide observational and bioinformatics evidence that even subtle alterations in specific proteins of the DNA repair pathways may contribute to CRC susceptibility and clinical outcome.

## 1. Introduction

Colorectal cancer (CRC) is the third most common malignancy and the second leading cause of cancer death worldwide [1]. In Europe, the highest incidence rates are reported in Eastern and Central European countries, such as the Czech Republic and Austria [2]. CRC represents a multifactorial disease associated with several genetic and environmental factors [3].

The prognosis for patients with CRC is heavily dependent on stage at diagnosis; the five-year survival rate is up to 90% for stage I, but only <15% for stage IV [4]. Over half of the cases are diagnosed at an advanced stage of disease (III and IV), with treatment usually involving complete primary tumor resection and appropriate chemotherapy. While the treatment can reduce the risk of relapse and increase patients’ survival, it can also cause severe side effects and impair quality of life [5]. The differences in medication response are considerably affected by individual inherited genetic susceptibility. Current approaches to choose and implement chemotherapy regimens for CRC patients are primarily determined by tumor staging and histopathological examination. Developing prognostic and predictive biomarkers based on a personal genetic background would greatly aid the selection of an optimal treatment by oncologists, so as to improve clinical outcome for each patient.

Genetics plays a key role in predisposition to CRC, its initiation, and progression [6]. Several studies provided evidence that single nucleotide polymorphisms (SNPs) in DNA repair genes could alter DNA repair function, modulate its capacity, and thus induce genetic instability or unregulated cell growth and cancer [7,8,9]. In the last decade, while association studies (including genome-wide) have identified multiple SNPs involved in CRC susceptibility, none have been validated as biomarkers for clinical use [10,11,12,13,14]. Furthermore, most of the anticancer agents are targeted to induce DNA damage, which overwhelms the cellular DNA repair capacity and thus leads to apoptosis. The most affected are the rapidly dividing cells, such as cancer cells. Treatment efficacy is therefore influenced by the DNA repair capacity of cancer cells, and the differences in treatment response might be affected by the inherited variations of genes encoding DNA repair enzymes [15].

In this study, we hypothesized that SNPs causing amino acid substitution (non-synonymous SNPs—nsSNPs) in DNA repair genes that are known to be involved in maintaining genome stability (cancer prevention) and in chemotherapy response (cancer treatment), may influence CRC susceptibility and modulate the clinical outcome after cancer diagnosis. We evaluated the association of 16 nsSNPs in 12 DNA repair genes with CRC risk, post-diagnosis survival, and therapy outcomes in a discovery set of 1832 patients and 1172 controls from the Czech Republic and in an independent replication set comprising 950 patients and 820 controls from Austria.

## 2. Results

### 2.1. SNP Selection

In total, sixteen nsSNPs in 12 genes passed the selection criteria and were successfully genotyped and analyzed in the Czech cohort (Table 1). The same nsSNPs were analyzed in the replication Austrian cohort, except for two nsSNPs (*FAAP24* rs3816032 and *MUS81* rs545500), where the genome-wide association study (GWAS) data were not available.

### 2.2. Case-Control Study

The characteristics of the study participants are shown in Table 2. Compared with controls, CRC cases in the Czech cohort had a slightly higher prevalence of male individuals, and were more likely to be older, to smoke, to have diabetes mellitus, and a positive family history of CRC. In the Austrian cohort, CRC cases were more often males and smokers.

For all of the SNPs, the distribution of the genotypes within the studied genes in controls was in agreement with the Hardy–Weinberg equilibrium. The SNPs significantly associated with CRC risk are presented in Table 3.

**Czech cohort.** The carriers of the *TC* genotype in *EME1* rs12450550 had an associated increased CRC risk (*TC* vs. *TT*; odds ratio (OR) 1.19; 95% confidence interval (CI) 1.00–1.40; *p* = 0.05), with the same tendency observed for the presence of the variant C allele in the dominant model (*TC+CC* vs. *TT*; OR 1.19; 95% CI 1.02–1.40; *p* = 0.03). However, this association should be considered cautiously due to the low frequency of the variant *CC* genotype in this study group. The variant *AA* genotype in *REV3L* rs3204953 was associated with an increased risk of CRC in the codominant and recessive model (*AA* vs. *GG*; OR 2.32; 95% CI 1.27–4.25; *p* = 0.006; and *AA* vs. *GG*+*GA*; OR 2.28; 95% CI 1.24–4.17; *p* = 0.008). After stratification according to the tumor site, the association was similar in colon cancer patients (*AA* vs. *GG*; OR 2.59; 95% CI 1.36–4.91; *p* = 0.004; and *AA* vs. *GG+GA*; OR 2.52; 95% CI 1.33–4.77; *p* = 0.005). By considering multiple testing correction using the Benjamini–Hochberg false discovery rate (FDR) measure, only the association for rs3204953 in *REV3L* remained significant (*q* = 0.01).

**Austrian cohort.** The association of the SNP genotypes with CRC risk was observed only for the rectal cancer patients. Carriers of the *CT* genotype in *POLQ* rs1381057 were at an increased risk of the disease (*CT* vs. *CC*; OR 1.32; 95% CI 1.01–1.74; *p* = 0.04), with the same tendency observed in the presence of the variant T allele in the dominant model (*CT*+*TT* vs. *CC*; OR 1.34; 95% CI 1.04–1.74; *p* = 0.03). The variant *GG* genotype in *REV1* rs3087399 was associated with a decreased risk of rectal cancer in the codominant and recessive model (*GG* vs. *AA*; OR 0.13; 95% CI 0.02–0.96; *p* = 0.05; and *GG* vs. *AA+AG*; OR 0.12; 95% CI 0.02–0.90; *p* = 0.04). None of the associations remained significant after the Benjamini–Hochberg correction.

### 2.3. Survival Analyses

In total, 1832 Czech and 950 Austrian CRC cases were included in the survival analyses. In the univariate assessment, several covariates were associated with survival, including established prognostic factors such as male sex, higher age, smoking habit, and cancer stage, which were associated with decreased patients’ survival and increased risk of recurrence (Table 4).

**Czech cohort.** Overall, no SNPs were associated with either the overall survival (OS) or event free survival (EFS). However, after stratification according to tumor localization, nominally significant associations were detected for six SNPs in the univariate assessment (Table S1). In colon cancer patients, two SNPs were associated with increased EFS (rs3816032 and rs2283432; *p* = 0.02 for either variant genotype). In rectal cancer patients, one SNP was associated with an increased EFS (rs7689099; *p* = 0.02), and three with decreased OS or EFS (rs545500, rs3218649, and rs3087386; *p* = 0.02, 0.02, and 0.03, respectively).

**Austrian cohort.** Four SNPs were associated with either OS or EFS (Table S1). Three SNPs were observed in association with increased OS or EFS in CRC patients (rs12450550, rs2283432, and rs3204953; *p* = 0.03, *p* = 0.02, and *p* = 0.02, respectively). Rs3087386 was found to be significantly associated with decreased OS and EFS in colon cancer patients (*p* = 0.02).

### 2.4. Survival and Therapy

To examine the association of SNPs with the therapy outcome, we further stratified patients according to the treatment received into the following three separate groups: (1) CRC patients receiving no treatment or (2) patients receiving a 5-Fluorouracil (5-FU) regimen without or (3) in combination with oxaliplatin. The group of patients treated with a combination of 5-FU and oxaliplatin was investigated separately, because the latter drug induces a different type of DNA damage compared to 5-FU alone, and thus different DNA repair pathways and genes may be involved [16,17]. The univariate model for survival and therapy showed several genotypes nominally significantly associated with OS or EFS (detailed description in supplementary text and Tables S2, S3, and S4).

### 2.5. Classification and Regression Tree Survival Analysis

**Figure 1:**
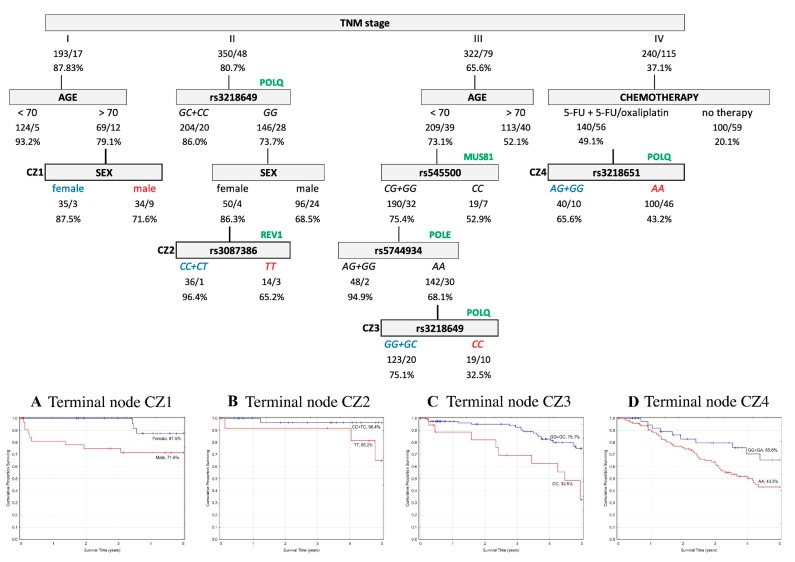
Overall survival (OS) classification and regression tree analysis of colorectal cancer patients from the Czech Republic. Numbers under each node indicate the total number of cases in the subcategory/number of events and percentage of patients with five-year OS. Terminal nodes are bordered in bold, and the corresponding Kaplan–Meier curves are shown underneath. (**A**) Terminal node CZ1; (**B**) Terminal node CZ2; (**C**) Terminal node CZ3; (**D**) Terminal node CZ4.

**Figure 2:**
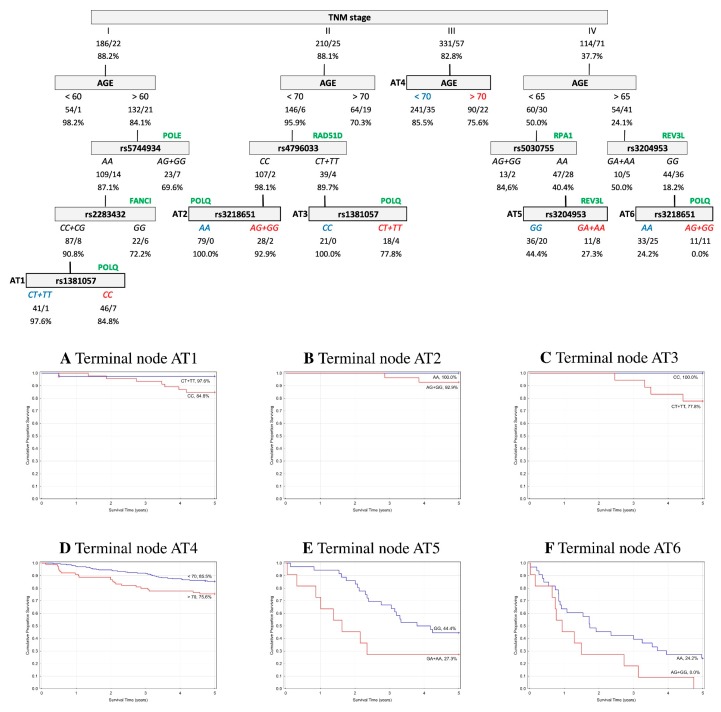
Overall survival (OS) classification and regression tree analysis of colorectal cancer patients from Austria. Numbers under each node indicate the total number of cases in the subcategory/number of events, and the percentage of patients with five-year OS. Terminal nodes are bordered in bold, and the corresponding Kaplan–Meier curves are shown underneath. (**A**) Terminal node AT1; (**B**) Terminal node AT2; (**C**) Terminal node AT3; (**D**) Terminal node AT4; (**E**) Terminal node AT5; (**F**) Terminal node AT6.

**Figure 3:**
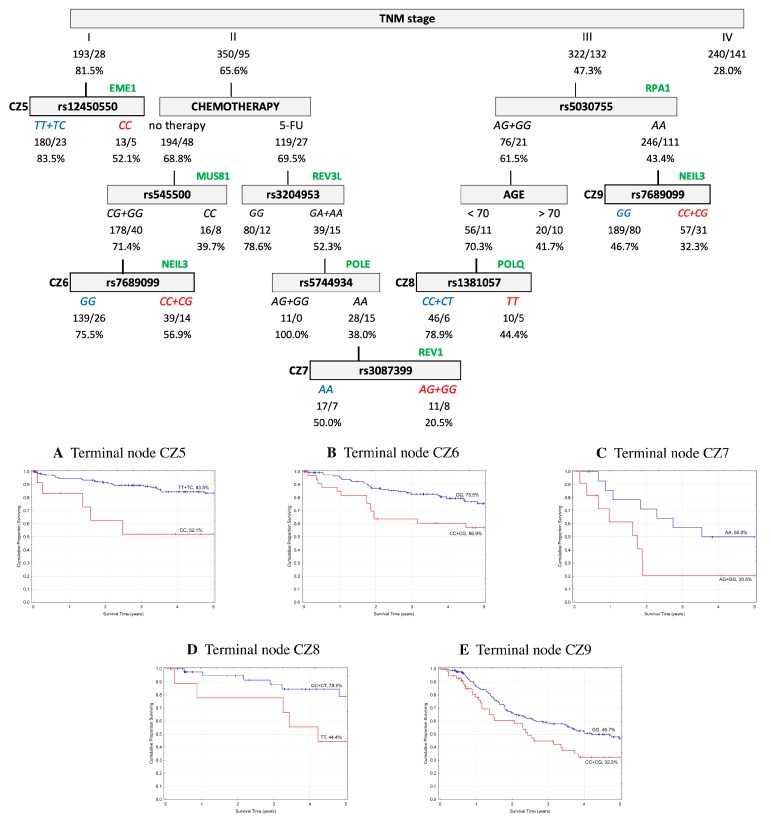
Event free survival (EFS) classification and regression tree of colorectal cancer patients from the Czech Republic. Numbers under each node indicate the total number of cases in the subcategory/number of events, and the percentage of patients with five-year EFS. Terminal nodes are bordered in bold, and the corresponding Kaplan–Meier curves are shown underneath. (**A**) Terminal node CZ5; (**B**) Terminal node CZ6; (**C**) Terminal node CZ7; (**D**) Terminal node CZ8; (**E**) Terminal node CZ9.

**Figure 4:**
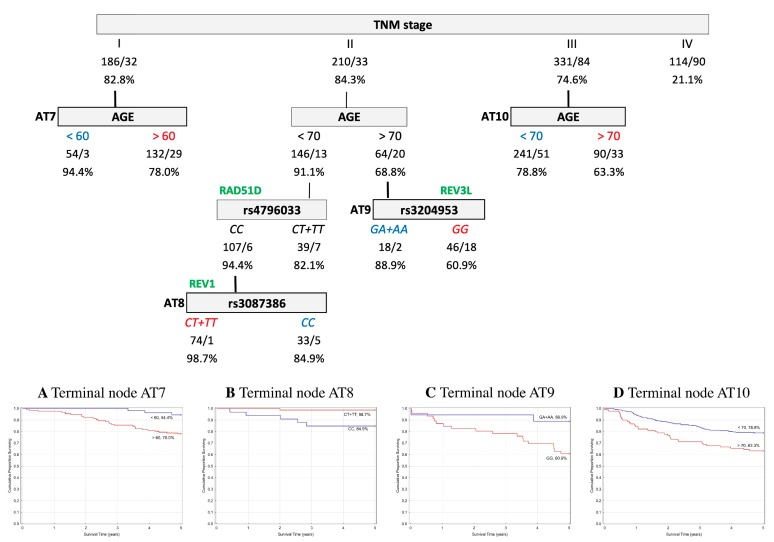
Event free survival (EFS) classification and regression tree of colorectal cancer patients from Austria. Numbers under each node indicate the total number of cases in the subcategory/number of events and the percentage of patients with five-year EFS. Terminal nodes are bordered in bold, and the corresponding Kaplan–Meier curves are shown underneath. (**A**) Terminal node AT7; (**B**) Terminal node AT8; (**C**) Terminal node AT9; (**D**) Terminal node AT10.

In order to assess the prognostic utility of the investigated DNA repair gene polymorphisms, the interactive effects of genotypes and clinico-pathological parameters in association with five-year OS and EFS were explored using a classification and regression tree (CART) analysis. Only patients with complete data for all of the parameters described in the Material and Methods were included in the analysis (*n* = 1105 (60%) for the Czech CRCs, and *n* = 841 (88%) for the Austrian CRCs). The results indicated that the tumor–node–metastasis (TNM) stage was chosen as the initial optimal split factor for predicting both OS and EFS in both of the cohorts (Figure 1, Figure 2, Figure 3 and Figure 4).

#### 2.5.1. Overall Survival

**Czech cohort.** The five-year OS analysis resulted in four terminal nodes. Variables determining the structure of the tree included TNM stage, age, sex, chemotherapy, and five SNPs—rs3087386, rs3218649, rs3218651, rs545500, and rs5744934. Among the stage I CRC patients, the subsequent split showed interactions between age and sex. In stage II, the carriers of *GC*+*CC* genotypes in *POLQ* rs3218649 were associated with a better prognosis. However, the *GG* genotype in females showed almost similar OS prognosis and an even better prognosis when in combination with *CC*+*CT* genotypes in *REV1* rs3087386 (*CC*+*CT* 96.4% vs. *TT* 65.2%). In stage III, the subsequent split was age, which was seen to interact with *GG*+*GC* genotypes in *MUS81* rs545500 and *AG*+*GG* genotypes in *POLE* rs5744934 (*AG*+*GG* 94.9% vs. *AA* 68.1%). The *AA* genotype of *POLE* gene further interacted with *POLQ* rs3218649 (*GG*+*GC* genotypes 75.1% vs. *CC* genotype 32.5%). In stage IV, chemotherapy was the next most significant factor and the level of OS increased when in combination with the rs3218651 variant in *POLQ* gene (*AG*+*GG* 65.6% vs. *AA* 43.2%). The structure of the tree and corresponding survival curves from terminal nodes are presented in Figure 1.

**Austrian cohort.** The final tree structure contained six terminal nodes and included nine variables (age, TNM stage, and seven SNPs—rs1381057, rs2283432, rs3204953, rs3218651, rs4796033, rs5030755, and rs5744934). Among CRC patients at stage I, age as the subsequent split showed interactions with the *AA* genotype in *POLE* rs5744934 and *CC+CG* genotypes in *FANCI* rs2283432. The interaction was concluded by *CT+TT* genotypes in *POLQ* rs1381057 (*CT+TT* 97.6% vs. *CC* 84.8%). In stage II, age was the next most significant factor, and the level of OS increased when in combination with the *CC* genotype in *RAD51D* rs4796033 and the *AA* genotype in *POLQ* rs3218649 (*AA* 100% vs. *AG+GG* 92.9%). Carriers of the *CT+TT* genotype in *RAD51D* rs4796033 showed a better prognosis in combination with the *CC* genotype in *POLQ* rs1381057 (*CC* 100% vs. *CT+TT* 77.8%). In stage III, age was the most significant factor for OS. In stage IV, age was further associated with three SNPs (*RPA1* rs5030755 combined with *REV3L* rs3204953 as a terminal node: *GG* 44.4% vs. *GA+AA* 27.3%; and *REV3L* rs3204953 combined with *POLQ* rs3218651 as a terminal node: *AA* 24.2% vs. *AG+GG* 0%). The structure of the tree and corresponding survival curves from terminal nodes are presented in Figure 2.

#### 2.5.2. Event-Free Survival

**Czech cohort.** Regarding the five-year EFS, the final tree structure contained five terminal nodes and included 11 variables (age, TNM stage, chemotherapy, and eight SNPs—rs12450550, rs1381057, rs3087399, rs3204953, rs5030755, rs545500, rs5744934, and rs7689099). Among CRC patients at stage I, the subsequent split was for *EME1* rs12450550 (*TT*+*TC* 83.5% vs. *CC* 52.1%). In stage II, chemotherapy was the first split, when patients with no treatment and those with 5-FU-based therapy without oxaliplatin showed almost the same prognosis level. Patients without treatment had a better prognosis when associated with *CG*+*GG* genotypes in *MUS81* rs545500 in combination with the *GG* genotype in *NEIL3* rs7689099 (*GG* 75.5% vs. *CC*+*CG* 56.9%). Patients treated only with 5-FU had a better prognosis when in association with the *GG* genotype in *REV3L* rs3204953 (*GG* 78.6% vs. *GA*+*AA* 52.3%). On the other hand, the negative association for rs3204953 with the prognosis level was further worsened by the *AA* genotype in *POLE* rs5744934 and *AG*+*GG* genotype in *REV1* rs3087399 (*AA* 50.0% vs. *AG*+*GG* 20.5%). In stage III, the subsequent split *AG*+*GG* genotype in *RPA1* rs5030755 was seen to interact with patients under 70 years of age and the *CC*+*CT* genotype in *POLQ* rs1381057 (*CC*+*CT* 78.9% vs. *TT* 44.4%). Patients with the *AA* genotype for rs5030755 were further associated with a worse prognosis level in combination with the wild type allele C in *NEIL3* (*GG* 46.7% vs. *CC*+*CG* 32.3%). The structure of the tree and corresponding survival curves from terminal nodes are presented in Figure 3.

**Austrian cohort.** The final tree structure contained four terminal nodes determined by five variables (age, TNM stage, and three SNPs—rs3087386, rs3204953, and rs4796033). In stage I, a better EFS prognosis was shown within patients under 60 years of age. In stage II, the subsequent split was age, which was seen to interact with the *CC* genotype in *RAD51D* rs4796033 and *CT*+*TT* genotypes in *REV1* rs3087386 (*CT*+*TT* 98.7% vs. *CC* 84.9%). Furthermore, *GA+AA* genotypes in *REV3L* rs3204953 showed a better EFS prognosis in stage II patients over the age of 70 (*GA+AA* 88.9% vs. *GG* 60.9%). In stage III, the subsequent split showed an interaction with age only. The structure of the tree and corresponding survival curves from terminal nodes are presented in Figure 4.

## 3. Discussion

DNA repair has an essential role in maintaining genome integrity and preventing carcinogenesis. Amino acid alterations by nsSNPs in DNA repair genes can cause changes to the function or level of the coded proteins, resulting in abrogated DNA repair, which in combination with continuous endogenous DNA damage over time could lead to genomic damage and carcinogenesis [7,18]. In the present study, we sought to identify associations between 16 potentially functional genetic polymorphisms in 12 DNA repair genes with CRC risk, patients’ survival, and response to chemotherapy in Czech and Austrian cohorts. To our knowledge, no similar studies have previously examined these selected SNPs in relation to CRC susceptibility and clinical outcomes after diagnosis.

In the discovery set from the Czech Republic, the results showed an association between the variant *AA* genotype in *REV3L* rs3204953 and an increased risk of CRC. *REV3L* encodes a catalytic subunit of an error-prone DNA polymerase ζ, whose involvement in both double strand break (DSB) repair and translesion synthesis (TLS) pathways may explain why it is the only known specialized DNA polymerase reducing spontaneous tumor development [19,20]. DSBs, i.e. breaks in both DNA strands, are one of the most cytotoxic lesions for genetic integrity, and if not adequately repaired, DSB can result in mutagenic events or cell death [21]. TLS is a DNA damage tolerance process that allows cells to continue replication past DNA templates containing bulky lesions without resulting in stalled replication forks and therefore preventing DNA strand breaks.

Disrupted REV3L in cancer cell lines showed the importance of accurately regulated *REV3L* expression, when its inhibition induced DNA damage and growth arrest in cancer cells, whereas overexpression led to increased spontaneous mutation rates [22]. Expression levels of this polymerase have also been linked to sensitivity to chemotherapy. While defects in the protein resulted in an increased sensitivity to therapy in multiple tumor cell lines, its overexpression induced increased therapy resistance [23,24,25]. Furthermore, a decreased expression of *REV3L* has also been reported in tumor compared with the adjacent non-malignant tissue in colon cancer [26,27].

An association of rs3204953 was observed with a higher risk of breast cancer in a Swedish cohort, however, the results were not replicated in a Polish cohort [28]. Other genetic variants in *REV3L* have been found to be associated with both disease development risk and patients’ survival for different tumor types, such as breast cancer, stomach cancer, and CRC [28,29,30]. None of the other associated SNPs were found in linkage disequilibrium with rs3204953 examined here.

In addition to the in silico predictions of the F-SNP database of the deleterious nature of the rs3204953 SNP for *REV3L* protein function, we also used web-servers ELASPIC and DUET to assess the energetic impact of the amino acid change. In ELASPIC, the valine to isoleucine substitution was predicted to decrease the protein stability, resulting in a protein favoring an unfolded state (as the Gibbs free energy of folding for the domain affected by the SNP is changed by ∆∆*G* = 1.97).

Regarding the clinical outcome, results of the Czech five-year EFS CART analysis showed that rs3204953 in *REV3L* was chosen as the optimal split for the CRC stage II patients receiving 5-FU-based chemotherapy. This finding indicates its possible use in personalized treatment strategies by identifying CRC stage II patients who are likely to benefit from adjuvant therapy.

Despite the promising results in the Czech population, an association of *REV3L* SNP with CRC risk could not be confirmed in the Austrian replication set. However, *REV3L* emerged several times as the optimal split in the Austrian CART analyses as well. Thus, according to all of the available data, we suggest that the *REV3L* gene may impact CRC susceptibility, survival, and therapy outcomes and warrants further investigation.

In survival CART analyses, TNM stage and age were shown as the most significant prognostic factors in both of the study cohorts. Apart from these clinico-pathological factors, we observed significant associations of several nsSNPs with patients’ survival and clinical outcomes. However, a few of these were shown as significant more than once in the CART analysis, suggesting their potentially greater relevance on patients’ survival. For example, *POLQ* gene polymorphisms appeared four times as the optimal split factor in the Czech CART analyses (rs1381057, rs3218649 twice, and rs3218651) and four times in the Austrian CART analyses (rs1381057 twice and rs3218651 twice). Polymerase θ encoded by *POLQ* is an error-prone polymerase with a similar role to polymerase ζ, and is involved in the base excision repair (BER) and DSB repair [31]. In addition to DNA repair, this polymerase also plays a crucial role in TLS [32].

The expression of polymerase θ is tightly regulated. A complementary body of literature reported an upregulation of *POLQ* in different tumor tissues (breast cancer, non-small cell lung cancer, oral squamous cell carcinoma, stomach cancer, and CRC), and this overexpression acted as a strong prognostic factor [33,34,35,36].

Strikingly, at least nine polymorphisms out of 23 known SNPs in the human *POLQ* gene are predicted to alter protein function [32]. Several *POLQ* SNPs have also been associated with a risk of different tumors, such as breast cancer, esophageal cancer, and Non-Hodgkin’s Lymphoma [28,37,38,39,40]. While only some of the breast cancer studies included rs3218649, no significant association was detected [28,38,40] and none of the other associated SNPs were found in the linkage disequilibrium with our selected SNPs.

The abovementioned studies highlighted the significance of adequate *POLQ* functioning and regulation for tumor suppression. Furthermore, the protein stability prediction for rs1381057 by ELASPIC estimated a change of the Gibbs free energy to ∆∆*G* = 1.65, suggesting that the substitution of glutamine to arginine decreases the altered protein stability. Unfortunately, we could not perform a protein stability prediction of rs3218649 and rs3218651 by ELASPIC, as these SNPs do not fall within the domain boundaries required by the software.

Another SNP, rs7689099 in *NEIL3* gene, emerged twice in the Czech five-year EFS CART analysis as the optimal split factor after the TNM stratification, suggesting its significance in patients´ survival. The *NEIL3* encodes a DNA glycosylase, playing an important role in the first step of the BER pathway [41]. The process of eliminating damaged nucleotides by BER is crucial to evade mutations at these sites, which is likely to aid tumor suppression [42].

The upregulation of *NEIL3* appears to be involved in the maintenance of cancer cell growth or the progression of malignancy. Significantly elevated expression levels in tumors, compared to corresponding non-malignant tissues, were reported in 20 cancer sites, including CRC [43,44]. The overexpression was further observed in association with the progression of primary melanoma to distant metastasis [45].

Sequence variability in different DNA glycosylases have been proposed as susceptibility factors for different malignancies [46]. Specifically, *NEIL3* SNPs were associated with the risk of glioma, prostate, and thyroid cancer [47,48,49], with rs7689099 being associated with a reduced risk of differentiated thyroid carcinoma and prostate cancer [47,49]. None of the other associated SNPs were found in linkage disequilibrium with rs7689099.

As rs7689099 in *NEIL3* gene does not fall within the domain boundaries, we could not use ELASPIC protein stability prediction. Again, the association of *NEIL3* SNP with the survival of CRC patients was not replicated in the Austrian sample set. However, considering the available data, we suggest that the variation of the *NEIL3* gene also has relevance for CRC susceptibility, survival, and therapy outcome.

In agreement with the in silico predictions about the functionality of the SNPs, we observed several significant associations of different genetic variants with survival and clinical outcomes of CRC patients both from the Czech Republic and Austria. However, we were not able to confirm the particular associations of individual SNPs between the discovery and the replication set. One might argue that the failure to replicate the association results might be due to differential gene–environmental interactions, and the differences in the clinical composition between the case-control populations of the discovery and the replication set (Table 2). Furthermore, it is also possible that other factors might have biased the results, for example earlier CRC detection in Austrian patients thanks to a better general awareness of the disease and a high standard medical care. This assumption is supported by the results in five-year OS CART analyses for stage III patients, where we observed a substantial difference between survival in the Czech and Austrian patients (65.6% vs. 82.8%). Our conclusion was based on the fact that CRC stage III is further divided into three more separate categories (IIIA, IIIB, and IIIC) according to the extent to which cancer has spread (i.e., number of lymph nodes affected). The survival rate then significantly decreases with the disease advancement. For example, in colon cancer patients, the survival rate for stage IIIA is about 90%, for stage IIIB 72%, and for stage IIIC only about 53% [50]. The strengths of the present work include the recruitment of a considerable number of cases and controls at the same centers, homogeneous for their ancestry (all Caucasian from the Czech Republic and Austria), and clinically well-defined (follow-up data collected by the same physicians), thus minimizing any possible population bias.

In conclusion, this is the first study to evaluate the association of genetic variants in DNA repair genes, selected by likely functional relevance with CRC. We identified several nsSNPs potentially affecting either CRC susceptibility or patients’ survival. Our data provide observational evidence of the potential role of nsSNPs in CRC pathogenesis, and suggest that even subtle alterations in the specific proteins that function in DNA repair pathways may lead to inaccurate DNA repair, and thus contribute to carcinogenesis.

Due to the lack of replication of significant associations, further studies on independent populations are warranted. This is underlined by the involvement of the same DNA repair genes in both Czech and Austrian CRC populations. Moreover, it is important to functionally characterize these candidate genetic variants, and to find biological mechanisms underlying the associations in order to assess these nsSNPs as prognostic and/or predictive biomarkers in CRC. Potential clinical uses are to help define individual CRC risk and tailor disease management based on the unique molecular profile of each patient.

## 4. Material and Methods

### 4.1. SNP Selection and In Silico Analysis of Functional Relevance and Conservation

From the complete list of DNA repair genes available online (http://sciencepark.mdanderson.org/labs/ wood/DNA_Repair_Genes.html, March 2014 version), all of the genes involved in repairing DNA damage caused by 5-FU or oxaliplatin were retrieved, as these are common chemotherapeutic treatment regimens for CRC.

In total, 106 genes of BER, nucleotide excision repair (NER), and DSB (including interstrand cross-links repair (ICL), fanconi anemia (FA), and TLS pathways) were searched for nsSNPs in the freely available F-SNP database [51]. The database also provides integrated information about possible effects of the base change on the coded protein, and thus helps to identify nsSNPs with a potential pathological effect on human health. The F-SNP data are obtained from several genomic databases, like SIFT, PolyPhen2, SNPeffect, and SNPs3D. The variants predicted as deleterious or damaging were further studied.

Selected relevant nsSNPs were then filtered for a MAF >10% in European populations to provide sufficient study power with the size of our case-control study, in order to uncover moderate genetic effects. The information was primarily derived from the Ensembl 2015 database—1000 Genomes Project Phase 3, EUR population (https://www.ensembl.org/index.html). Whenever this was not possible, other reference populations were considered (i.e., HAPMAP CEU population).

The SNPs with the required MAF were tested for linkage disequilibrium (LD) with the data from HapMap (v. 3, release R2 in the CEU population, ftp://ftp.ncbi.nlm.nih.gov/hapmap/). The 38 identified nsSNPs were further searched within the Genetic Association Database (http://geneticassociationdb.nih.gov, accessed on 9 January 2014). From these, sixteen nsSNPs were already investigated elsewhere in relation to CRC, and therefore were excluded from this study.

The 22 remaining nsSNPs were tested by comparative genomics to evaluate the probability that the nucleotide is located in an evolutionary conserved position or within a constrained element, using the Genomic Evolutionary Rate Profiling GERP++ RS (Rejected Substitutions) score. An element with a GERP++ RS score >800 defines ultra-conserved regions among mammals. SiPhy evaluates the conservation of the motif around the SNPs.

**Figure 5:**
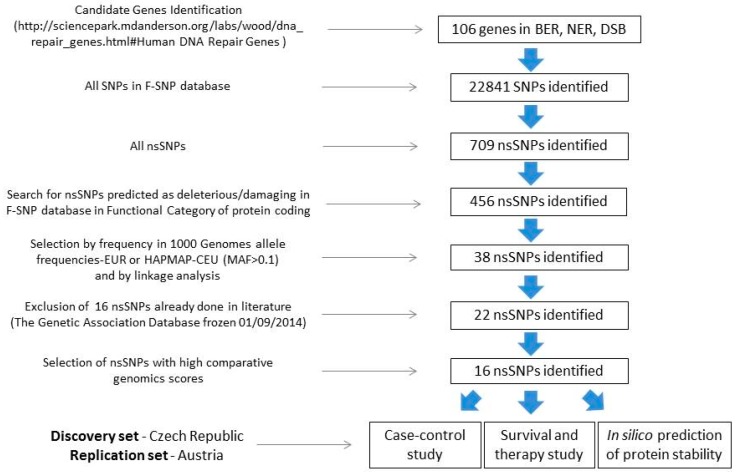
Workflow strategy for the selection and analyses of functional non-synonymous polymorphisms in DNA repair genes. BER—base excision repair; NER—nucleotide excision repair; DSB—double strand break repair; SNP—single nucleotide polymorphism; nsSNP—non-synonymous SNP; MAF—minor allele frequency.

After this selection, sixteen nsSNPs in 12 DNA repair genes complied with the required selection criteria. The workflow for the selection is depicted in Figure 5.

To evaluate the stability of the final protein affected by nsSNP, we further utilized web-server tools ELASPIC and DUET to assess the energetic impact of the amino acid change (http://elaspic.kimlab.org/ and http://biosig.unimelb.edu.au/duet/stability). The main output is the predicted variation in the Gibbs free energy (ΔΔ*G*) of folding and/or binding for every domain affected by the SNP.

### 4.2. Study Populations and Data Collection

Patients included in the study were newly diagnosed histologically confirmed individuals with sporadic CRC. The exclusion criteria were as follows: (1) hereditary CRC forms (Lynch syndrome and familial adenomatous polyposis) and (2) a personal history of previous malignant disease. Personal data, such as date of birth, sex, lifestyle habits, body mass index (BMI), diabetes mellitus, and family/personal history of cancer, were obtained using a structured questionnaire in order to determine potential risk factors for CRC. For all subjects, clinical data including tumor-related parameters, such as the location of the tumor, International Union Against Cancer (UICC) TNM stage system, degree of tumor differentiation, and adjuvant chemotherapy treatment details, were collected, along with information about distant metastasis, relapse, and date of death.

Patients were divided into three subgroups according to the therapy received. The first group of patients did not receive any adjuvant chemotherapy after surgery. The second group of patients received a 5-FU-based adjuvant regimen as a postoperative therapy (based either on a Mayo, a simplified DeGramont, or a Xeloda regimen). The third group of subjects received adjuvant 5-FU treatment combined with oxaliplatin (based either on a FOLFOX or a XELOX regimen).

The study was approved by the local ethics committee of each participating hospital, and written informed consent to participate in the study and to approve the use of their biological samples for genetic analyses was obtained from all patients, according to the 1964 Helsinki declaration.

#### 4.2.1. Discovery Set—Czech Republic

Patients (*n* = 1832) were recruited at several oncological and gastroenterological departments of different hospitals all over the Czech Republic from September 2003 to January 2014. The last update of the patients’ follow-up for this study was in December 2015. Characteristics of the study participants are shown in Table 2 (partially described in the literature [52,53].

The control group consisted of 659 healthy blood donors and 513 colonoscopy-negative controls, which were collected during the same time period as the cases. Healthy blood donor volunteers were recruited at the Faculty Hospital Kralovske Vinohrady in Prague and the Vojkov hospital. The group of colonoscopy-negative controls consisted of subjects admitted to the hospital gastroenterology departments who had negative colonoscopy results for malignancy or idiopathic bowel diseases. The reasons for undergoing the colonoscopy were as follows: (i) positive fecal occult blood test, (ii) hemorrhoids, (iii) abdominal pain of unknown origin, and (iv) macroscopic bleeding. All individuals were subjected to standard examinations so as to verify the health status for blood donation, and were cancer-free at the time of the sampling.

DNA was extracted from the peripheral blood lymphocytes using standard procedures. When blood was not available (for 690 cases), healthy colon/rectal tissue was used to obtain DNA by using the DNeasy Blood and Tissue Kit (Qiagen, Courtaboeuf, France). Genotyping was performed at LGC Genomics (Hoddesdon, Herts, UK), using the KASP™, a competitive allele-specific PCR genotyping system. For quality control purposes, duplicate samples (5% of the total numbers of samples) were repeated for each SNP. Two no-template controls were included in each plate. The genotype correlation between the duplicate samples was >98%. Two CRC cases were eliminated due to low genotyping rates.

#### 4.2.2. Replication Set—Austria

In the ongoing Colorectal Cancer Study of Austria (CORSA), over 13,000 participants comprising CRC cases (stages I–IV); adenomas; and population-based, colonoscopy-negative controls have been recruited since 2003, in cooperation with the province-wide screening program “Burgenland Prevention Trial of Colorectal Disease with Immunological Testing” (B-PREDICT). All inhabitants of the Austrian province Burgenland aged between 40 and 80 years are invited annually to participate in fecal immunochemical testing (FIT). FIT-positive tested individuals are offered a complete colonoscopy and are asked to participate in CORSA at the time of colonoscopy. Only the individuals with histologically confirmed sporadic CRC were included in this study.

Further CRC cases were recruited at multiple centers in Vienna, including the Medical University of Vienna (Department of Surgery), the Sozialmedizinisches Zentrum Süd, the Hospital Rudolfstiftung, and the Medical University of Graz (Department of Internal Medicine). The replication set comprised 950 CRC patients and 820 colonoscopy-negative controls from CORSA. The last update of the patients’ follow-up was performed in August 2018. Baseline characteristics of this cohort are presented in Table 2 , and the study has previously been described [54].

The genomic DNA isolation from peripheral blood was performed using the QIAamp DNA Blood Midi Kit, according to the manufacturer’s recommendations (Qiagen, Valencia, CA, USA), and was stored at −80 °C. Genotyping was performed using the population-optimized Axiom Genome-Wide CEU 1 Array (Affymetrix, Santa Clara, CA, USA). The arrays were processed at the Institute of Human Genetics, Helmholtz Center Munich, Germany, and genotype assignment was performed as described in Hofer et al. [54]. Data for two SNPs (*FAAP24* rs3816032 and *MUS81* rs545500) were not covered on the array, and therefore could not be included in further analyses.

### 4.3. Statistical Analysis

In controls, the genotype frequencies for each polymorphism were tested for deviation from the Hardy–Weinberg equilibrium, using a Pearson χ^2^-test (1 degree of freedom) with a type-I error threshold set at α = 0.05.

The association between nsSNPs and CRC risk was determined by logistic regression, and was calculated by estimating the ORs, and their 95% CIs were adjusted for age. The ancestral allele (evolutionary primal) was used as a reference. For all nsSNPs, co-dominant, dominant, and recessive models were calculated.

In this study, the outcome variables measured were OS and EFS. OS was defined as the time from the surgery to the date of death, or the date of last follow up (for the Czech cohort it was December 2015, for the Austrian cohort it was August 2018). EFS was defined as the time from surgery to the occurrence of distant metastasis, local recurrence, or death, whichever came first. The survival curves for OS and EFS were derived by the Kaplan–Meier log-rank test. The relative risk of death and recurrence was estimated as a hazard ratio (HR) with 95% CIs, using Cox regression (no covariates adjustment was applied).

A multivariate analysis, referred to as a CART [55], was used to assess the prognostic value of interactions between the standard clinico-pathological variables and the genetic variants in relation to their impact on five-year survival in CRC patients. The analysis constructs a set of decision rules that stratify the homogenous risk groups of the responsive variable. Splits for each variable were examined, and the variable (predictor) that provides the best or “optimal” split was selected. Each subgroup was further divided in the same manner. In the Czech sample set, CART was implemented using nine common clinical and pathological variables, including age, sex, smoking habit (non-smokers vs. smokers vs. ex-smokers), diabetes mellitus, positive family history of CRC, diagnosis (colon vs. rectal cancer), TNM stage, grade, and therapy (no therapy vs. 5-FU-based without oxaliplatin vs. 5-FU in combination oxaliplatin), and all examined nsSNPs. In the Austrian sample set, because the information for five of the variables (smoking habit, positive family history of CRC, grade, and nsSNPs rs3816032 and rs545500) were only available for a small number of patients, only six common clinico-pathological variables and 14 nsSNPs were implemented for the CART analysis of this cohort.

Statistical analyses were performed using SAS software (SAS Institute, Cary, NC, USA). Graphs were performed using SW STATISTICA (StatSoft, Inc., Tulsa, OK, USA). Multiple testing corrections were performed using the Benjamini–Hochberg FDR [56].

## Figures and Tables

**Table 1:** Selected single nucleotide polymorphisms (SNPs) in DNA repair genes, with a minor allele frequency of ^a^ > 0.10 in the European population.

Genomic Annotation							Functional Genomics					Comparative Genomics	
Gene ID	DNA Repair Pathway	UniProtKB	SNP ID	Base Change	Amino Acid Change	MAF in EUR ^a^	LD with Other SNPs Associated with CRC	LD within the Same Gene	F-SNP Prediction Result (on Protein Coding)	ELASPIC (∆∆*G*)	DUET (∆∆*G*)	Element GERP RS Score >800	SIPHYs
*EME1*	DSB	Q96AY2	rs12450550	T > C	Ile350Thr	0.24	no	no	deleterious	Destabilizing (Core 1.646)	Destabilizing (−3.002 Kcal/mol)		
*FAAP24*	DSB	Q9BTP7	rs3816032 ^b^	T > C	Ile192Thr	0.11	no	no	deleterious	Destabilizing (Core 1.133)	Destabilizing (−1.653 Kcal/mol)		X
*FANCI*	DSB	Q9NVI1	rs2283432	C > G	Cys742Ser	0.38	no	no	deleterious	NA	NA	X	X
*MUS81*	DSB	Q96NY9	rs545500 ^b^	C > G	Arg180Pro	0.33	no	no	deleterious	NA	NA		X
*NEIL3*	BER	Q8TAT5	rs7689099	C > G	Pro117Arg	0.12	no	no	deleterious	NA	NA	X	X
*POLE*	BER, DSB, NER	Q07864	rs5744934	A > G	Asn1396Ser	0.13	no	no	deleterious	NA	NA		X
*POLN*	DSB	Q7Z5Q5	rs2353552	C > A	Gln121His	0.13	no	no	deleterious	NA	NA		
			rs9328764	A > G	Arg425Cys	0.12	no	no	deleterious	NA	Destabilizing (−1.765 Kcal/mol)		X
*POLQ*	DSB	O75417	rs1381057	C > T	Gln2513Arg	0.33	no	no	deleterious	Destabilizing (Core 1.648)	NA		X
			rs3218649	C > G	Thr982Arg	0.39	no	no	deleterious	NA	NA	X	
			rs3218651	T > C	His1201Arg	0.15	no	no	damaging	NA	NA	X	
*RAD51D*	DSB	O75771	rs4796033	C > T	Arg165Gln	0.13	no	no	deleterious	Destabilizing (Core 1.843)	NA		X
*REV1*	DSB	Q9UBZ9	rs3087386	G > A	Phe257Ser	0.43	no	no	deleterious	NA	NA	X	
			rs3087399	A > G	Asn373Ser	0.12	no	no	deleterious	NA	Destabilizing (−0.596 Kcal/mol)	X	X
*REV3L*	DSB	O60673	rs3204953	G > A	Val2986Ile	0.17	no	no	deleterious	Destabilizing (Core 1.965)	NA	X	X
*RPA1*	BER, DSB, NER	P27694	rs5030755	A > G	Thr351Ala	0.10	no	no	deleterious	NA	Destabilizing (−1.037 Kcal/mol)		X

SNP—single nucleotide polymorphism; MAF—minor allele frequency; EUR—European population; LD—linkage disequilibrium; CRC—colorectal cancer; GERP—Genomic Evolutionary Rate Profiling; DSB—double strand break repair pathway; BER—base excision repair pathway; NER—nucleotide excision repair pathway; NA—not applicable; X—evolutionary conserved position. ^a^ https://www.ensembl.org/index.html. ^b^ Data for SNPs were not available in the Austrian cohort.

**Table 2:** Characteristics of the study populations.

		Czech Republic					Austria				
Variables		Controls	Cases	OR	95% CI	*p*-Value	Controls	Cases	OR	95% CI	*p*-Value
		No. (%)	No. (%)				No. (%)	No. (%)			
Sex	Female	478 (40.8)	696 (38.1)	Ref			353 (43.1)	389 (40.9)	Ref		
	Male	694 (59.2)	1133 (61.9)	1.20	1.02–1.40	0.03	467 (56.9)	561 (59.1)	1.39	1.09–1.78	0.01
Age (years)	<50	269 (22.9)	140 (9.2)	Ref			92 (11.2)	129 (13.5)	Ref		
	(50, 60]	546 (46.6)	433 (28.4)	1.52	1.20–1.94	0.0006	147 (17.9)	208 (21.9)	2.18	0.72–1.43	0.92
	(60, 70]	183 (15.6)	639 (42.0)	6.71	5.16–8.72	<0.0001	282 (34.4)	323 (34.0)	0.82	0.60–1.13	0.22
	>70	174 (14.9)	310 (20.4)	3.42	2.60–4.51	<0.0001	299 (36.5)	291 (30.6)	0.70	0.51–0.96	0.03
BMI	(18.5, 25]	93 (8.0)	358 (23.5)	Ref			189 (23.7)	296 (35.7)	Ref		
	<18.5	334 (28.5)	370 (24.3)	3.22	2.45–4.24	<0.0001	2 (0.3)	17 (2.0)	5.43	1.24–23.76	0.02
	(25, 30]	529 (45.1)	508 (33.4)	0.84	0.69–1.02	0.08	364 (45.6)	336 (40.5)	0.59	0.47–0.75	<0.0001
	>30	213 (18.4)	286 (18.8)	1.13	0.89–1.43	0.31	243 (30.4)	181 (21.8)	0.48	0.37–0.62	<0.0001
Smoking habit	No	638 (57.6)	769 (53.1)	Ref			447 (55.7)	251 (48.8)	Ref		
	Yes ^a^	470 (42.4)	679 (46.9)	1.33	1.13–1.56	<0.001	356 (44.3)	263 (51.2)	1.30	0.97–1.73	0.08
DM	No	555 (85.5)	1076 (80.4)	Ref			370 (82.8)	817 (86.0)	Ref		
	Yes	94 (14.5)	263 (19.6)	1.41	1.09–1.84	0.01	77 (17.2)	133 (14.0)	0.62	0.42–0.92	0.02
Family history of CRC	No	942 (89.3)	1103 (84.1)	Ref			NDA	NDA			
	Yes	113 (10.7)	209 (15.9)	1.65	1.28–2.12	<0.001	NDA	NDA			
Diagnosis	Colon		1192 (65.8)					586 (62.6)			
	Rektum		621 (34.2)					350 (37.4)			
tnm stage	I		277 (16.8)					188 (21.2)			
	II		498 (30.2)					227 (25.5)			
	III		491 (29.8)					354 (39.8)			
	IV		384 (23.3)					120 (13.5)			
Chemotherapy	None		795 (49.9)					389 (43.0)			
	5-FU		494 (31.0)					253 (28.0)			
	5-FU combined with oxaliplatin		303 (19.1)					262 (29.0)			

OR—odds ratio; CI—confidence interval; BMI—body mass index; DM—diabetes mellitus; CRC—colorectal cancer; TNM—tumor-node-metastasis; 5-FU—5-fluorouracil; NDA—no data available. Significant results are in bold. Numbers may not add up to 100% of available subjects because of missing data. ^a^ Ex-smokers included in smokers.

**Table 3:** Associations of SNPs in DNA repair genes with the risk of CRC and its major sub-sites (colon/rectum).

			All CRC Patients				Colon Cancer Patients				Rectal Cancer Patients				
Gene	Genotype	Controls ^a^	Cases ^a^	OR ^b^	95% CI	*p*-Value	Cases ^a^	OR ^b^	95% CI	*p*-Value	Cases ^a^	OR ^b^	95% CI	*p*-Value	HWE ^c^
SNP															*Χ*^2^, *p*-Value
Czech Republic															
*EME1* rs12450550	*TT*	678	815	Ref			526	Ref			284	Ref			1.07, 0.58
	*TC*	410	570	1.19	1.00–1.40	0.05	363	1.17	0.97–1.40	0.11	198	1.20	0.96–1.50	0.11	
	*CC*	73	108	1.24	0.90–1.70	0.20	64	1.15	0.80–1.65	0.46	41	1.38	0.91–2.09	0.13	
	*TC+CC*	483	678	1.19	1.02–1.40	0.03	427	1.16	0.97–1.39	0.10	239	1.23	0.99–1.52	0.06	
	*TT+TC*	1088	1385	Ref			889	Ref			482	Ref			
	*CC*	73	108	1.16	0.84–1.59	0.36	64	1.08	0.76–1.55	0.66	41	1.28	0.86–1.93	0.23	
*REV3L* rs3204953	*GG*	839	1049	Ref			666	Ref			371	Ref			4.68, 0.10
	*GA*	304	405	1.09	0.91–1.30	0.37	261	1.12	0.91–1.37	0.27	139	1.06	0.83–1.34	0.66	
	*AA*	15	43	2.32	1.27–4.25	0.006 *	30	2.59	1.36–4.91	0.004 *	13	1.97	0.92–4.22	0.08	
	*GA+AA*	319	448	1.14	0.96–1.36	0.13	291	1.19	0.98–1.44	0.08	152	1.10	0.87–1.39	0.42	
	*GG+GA*	1143	1454	Ref			927	Ref			510	Ref			
	*AA*	15	43	2.28	1.24–4.17	0.008 *	30	2.52	1.33–4.77	0.005 *	13	1.95	0.91–4.18	0.09	
Austria															
*POLQ* rs1381057	*CC*	372	413	Ref			267	Ref			134	Ref			1.49, 0.47
	*CT*	349	423	1.09	0.90–1.34	0.38	250	1.00	0.80–1.25	1.00	166	1.32	1.01–1.74	0.04	
	*TT*	99	114	1.05	0.77–1.42	0.76	65	0.93	0.65–1.32	0.68	49	1.40	0.94–2.08	0.10	
	*CT+TT*	448	537	1.08	0.90–1.31	0.40	315	0.98	0.80–1.22	0.89	215	1.34	1.04–1.74	0.03	
	*CC+CT*	721	836	Ref			517	Ref			300	Ref			
	*TT*	99	114	1.00	0.75–1.34	0.98	65	0.93	0.66–1.29	0.66	49	1.21	0.84–1.75	0.32	
*REV1* rs3087399	*AA*	593	673	Ref			414	Ref			243	Ref			0.02, 0.99
	*AG*	208	259	1.10	0.89–1.36	0.39	151	1.04	0.81–1.32	0.78	105	1.25	0.95–1.66	0.11	
	*GG*	19	18	0.83	0.43–1.60	0.58	17	1.27	0.65–2.48	0.48	1	0.13	0.02–0.96	0.05	
	*AG+GG*	227	277	1.08	0.87–1.32	0.50	168	1.06	0.83–1.34	0.65	106	1.16	0.88–1.53	0.30	
	*AA+AG*	801	932	Ref			565	Ref			348	Ref			
	*GG*	19	18	0.81	0.42–1.56	0.53	17	1.26	0.65–2.44	0.50	1	0.12	0.02–090	0.04	

OR—odds ratio; CI—confidence interval. Nominally significant results are in bold. Results that passed the Benjamini–Hochberg test for multiple comparisons are marked with an asterisk. ^a^ Numbers may not add up to 100% of subjects due to genotyping failure. All of the samples that did not give a reliable result in the first round of genotyping were retested in up to two additional rounds. Samples failing these procedures were omitted from the analysis. ^b^ Logistic regression analysis values are adjusted for age. ^c^ X^2^ and *p*-values for the deviation of the observed and of the numbers expected from the Hardy–Weinberg equilibrium (HWE) in the controls.

**Table 4:** Clinical characteristics significantly affecting overall survival (OS) and event free survival (EFS) in CRC patients with complete follow up.

		Czech Republic					Austria				
Variables		N ^a^	OS		EFS		N ^a^	OS		EFS	
			HR (95% CI)	*p*-Value	HR (95% CI)	*p*-Value		HR (95% CI)	*p*-Value	HR (95% CI)	*p*-Value
Sex	Female	696	Ref		Ref		389	Ref		Ref	
	Male	1133	1.47 (1.20–1.80)	0.0002	1.29 (1.09–1.52)	0.003	561	1.37 (1.03–1.83)	0.03	1.43 (1.11–1.83)	0.005
Age (years)	<50	149	Ref		Ref		129	Ref		Ref	
	(50, 60]	433	0.96 (0.62–1.50)	0.87	1.06 (0.76–1.49)	0.72	208	1.44 (0.77–2.69)	0.26	1.62 (0.99–2.65)	0.05
	(60, 70]	639	1.08 (0.71–1.65)	0.72	0.90 (0.65–1.25)	0.54	323	2.14 (1.21–3.79)	0.01	1.68 (1.05–2.68)	0.03
	>70	610	1.47 (0.97–2.24)	0.07	1.05 (0.76–1.46)	0.77	291	3.11 (1.77–5.47)	<0.0001	2.53 (1.60–4.00)	<0.0001
BMI	(18.5, 25]	434	Ref		Ref		296	Ref		Ref	
	<18.5	456	0.99 (0.77–1.27)	0.92	1.06 (0.86–1.32)	0.58	17	1.12 (0.41–3.07)	0.83	1.31 (0.57–3.00)	0.52
	(25, 30]	626	0.83 (066–1.06)	0.13	0.94 (0.77–1.15)	0.54	336	0.79 (0.55–1.12)	0.18	0.85 (0.63–1.15)	0.29
	>30	315	0.58 (0.43–0.80)	0.0008	0.83 (0.65–1.06)	0.13	181	1.13 (0.77–1.65)	0.54	1.04 (0.74–1.46)	0.83
Smoking habit	No	967	Ref		Ref		251	Ref		Ref	
	Yes ^b^	777	1.266 (1.049–1.529)	0.01	1.27 (1.08–1.48)	0.003	263	0.93 (0.65–1.32)	0.67	1.02 (0.75–1.39)	0.91
Stage	I	277	Ref		Ref		188	Ref		Ref	
	II	498	1.75 (1.10–2.80)	0.02	1.99 (1.41–2.82)	0.0001	227	1.00 (0.57–1.76)	1.00	0.90 (0.56–1.45)	0.67
	III	491	3.46 (2.22–5.39)	<0.0001	3.45 (2.47–4.83)	<0.0001	354	1.51 (0.92–2.45)	0.10	1.55 (1.03–2.32)	0.03
	IV	384	8.91 (5.78–13.74)	<0.0001	6.00 (4.30–8.38)	<0.0001	120	7.98 (4.95–12.88)	<0.0001	9.33 (6.21–14.02)	<0.0001
5FU-based chemotherapy	No	765	Ref		Ref		389	Ref		Ref	
	Yes	797	1.022 (0.84–1.24)	0.82	1.387 (1.18–1.63)	<0.0001	515	1.33 (0.99–1.78)	0.06	1.79 (1.38–2.32)	<0.0001

HR—hazard ratio; CI—confidence interval; BMI—body mass index. Significant results are in bold. ^a^ Numbers may not add up to 100% of the available subjects because of missing information. ^b^ Ex-smokers included in smokers.

## Supplementary Materials

Supplementary materials can be found at http://www.mdpi.com/1422-0067/20/1/97/s1.

## Abbreviations

5-FU	5-Fluorouracil
BER	Base excision repair
BMI	Body mass index
CART	Classification and regression tree analysis
CI	Confidence intervals
CRC	Colorectal cancer
DSB	Double strand break repair
EFS	Event-free survival
FA	Fanconi anemia
FDR	False discovery rate
GERP	Genomic evolutionary rate profiling
GWAS	Genome-wide association study
HRs	Hazard ratios
ICL	Interstrand cross-links repair
LD	Linkage disequilibrium
MAF	Minor allele frequency
NER	Nucleotide excision repair
nsSNP	Non-synonymous single nucleotide polymorphism
ORs	Odds ratios
OS	Overall survival
RS	Rejected substitutions
TLS	Translesion synthesis
TNM	Tumor–node–metastasis stage system
UICC	International union against cancer
