# Supplementary material for: Functional Polymorphisms in DNA Repair Genes Are Associated with Sporadic Colorectal Cancer Susceptibility and Clinical Outcome
Source: Int J Mol Sci. 2018 Dec 27;20(1):97. doi: 10.3390/ijms20010097 (PMC6337670; doi:10.3390/ijms20010097)
Supplement: Supplementary file 1 [file ijms-20-00097-s001.pdf]

Supplementary Table S1: SNPs associated with OS and EFS of patients in the pooled population and in groups stratified for tumor sites for Czech Republic and Austrian populations (Cox regression for adjusted estimates).

		Overall survival															*Event free survival*														
		All CRC patients					***Colon cancer patients***					***Rectal cancer patients***					***All CRC patients***					***Colon cancer patients***					***Rectal cancer patients***				
**Gene SNP**	**Genotype**	**N[a]**	**Events**	**HR**	**95% CI**	*p*-Value	**N[a]**	**Events**	**HR**	**95% CI**	*p*-Value	**N[a]**	**Events**	**HR**	**95% CI**	*p*-Value	**N[a]**	**Events**	**HR**	**95% CI**	*p*-Value	**N[a]**	**Events**	**HR**	**95% CI**	*p*-Value	**N[a]**	**Events**	**HR**	**95% CI**	*p*-Value
																	**Czech Republic**																
FAAP24	*TT*	1383	342	Ref			889	236	Ref			480	102	Ref			1383	500	Ref			889	335	Ref			480	159	Ref		
**rs3816032**	*TC*	384	93	0.92	0.73-1.16	0.46	260	57	0.77	0.57-1.02	0.07	120	35	1.33	0.91-1.96	0.14	384	134	0.90	0.75-1.09	0.29	260	80	0.75	0.59-0.96	**0.02***	120	53	1.29	0.95-1.76	0.11
	CC	25	9	1.43	0.74-2.77	0.29	20	7	1.25	0.59-2.66	0.56	5	2	2.09	0.52-8.47	0.30	25	12	1.36	0.77-2.42	0.29	20	9	1.20	0.62-2.33	0.59	5	3	1.90	0.61-5.97	0.27
	TC+CC	409	102	0.95	0.76-1.18	0.63	280	64	0.80	0.61-1.05	0.11	125	37	1.36	0.93-1.98	0.11	409	146	0.93	0.77-1.12	0.42	280	89	0.78	0.62-0.99	**0.04**	125	56	1.31	0.97-1.78	0.08
	TT+TC	1767	435	Ref			1149	293	Ref			600	137	Ref			1767	634	Ref			1149	415	Ref			600	212	Ref		
	CC	25	9	1.45	0.75-2.81	0.27	20	7	1.32	0.63-2.80	0.46	5	2	1.93	0.48-7.79	0.36	25	12	1.39	0.79-2.47	0.25	20	9	1.27	0.66-2.46	0.48	5	3	1.79	0.57-5.58	0.32
FANCI	*CC*	620	160	Ref			399	109	Ref			214	49	Ref			620	237	Ref			399	160	Ref			214	75	Ref		
**rs2283432**	*CG*	875	206	0.91	0.74-1.12	0.36	566	133	0.83	0.64-1.06	0.14	301	71	1.10	0.76-1.58	0.61	875	295	0.87	0.73-1.03	0.10	566	186	0.77	0.62-0.95	**0.02***	301	105	1.06	0.79-1.43	0.70
	GG	295	75	0.97	0.73-1.27	0.80	199	56	0.97	0.70-1.34	0.84	93	18	0.90	0.53-1.55	0.72	295	111	0.96	0.77-1.21	0.75	199	76	0.88	0.67-1.51	0.34	93	34	1.16	0.77-1.73	0.49
	CG+GG	1170	281	0.92	0.76-1.12	0.42	765	189	0.86	0.68-1.09	0.22	394	89	1.06	0.75-1.50	0.76	1170	406	0.89	0.76-1.05	0.16	765	262	0.80	0.66-0.97	**0.02**	394	139	1.08	0.82-1.43	0.58
	CC+CG	1495	366	Ref			965	242	Ref			515	120	Ref			1495	532	Ref			965	346	Ref			515	180	Ref		
	GG	295	75	1.02	0.80-1.31	0.85	199	56	1.08	0.81-1.45	0.60	93	18	0.86	0.53-1.41	0.56	295	111	1.05	0.86-1.29	0.63	199	76	1.02	0.80-1.31	0.87	93	34	1.13	0.78-1.62	0.53
MUS81	*CC*	171	44	Ref			106	30	Ref			62	14	Ref			171	62	Ref			106	42	Ref			62	20	Ref		
**rs545500**	*CG*	761	175	0.80	0.58-1.12	0.19	488	123	0.82	0.55-1.22	0.33	266	50	0.73	0.41-1.33	0.31	761	267	0.91	0.69-1.20	0.51	488	183	0.90	0.64-1.25	0.52	266	82	0.92	0.56-1.50	0.73
	GG	847	217	0.92	0.66-1.27	0.60	562	140	0.84	0.57-1.24	0.38	277	74	1.08	0.61-1.92	0.78	847	305	0.97	0.74-1.27	0.81	562	189	0.82	0.59-1.15	0.25	277	111	1.29	0.80-2.07	0.30
	CG+GG	1608	392	0.86	0.63-1.18	0.34	1050	263	0.83	0.57-1.21	0.33	543	124	0.91	0.51-1.57	0.73	1608	572	0.94	0.72-1.22	0.65	1050	372	0.86	0.62-1.18	0.34	543	193	1.10	0.69-1.74	0.69
	CC+CG	932	219	Ref			594	153	Ref			328	64	Ref			932	329	Ref			594	225	Ref			328	102	Ref		
	GG	847	217	1.10	0.91-1.33	0.31	562	140	0.99	0.79-1.24	0.92	277	74	1.39	0.99-1.94	**0.05**	847	305	1.04	0.89-1.22	0.61	562	189	0.90	0.74-1.09	0.27	277	111	1.38	1.05-1.81	**0.02***
NEIL3	*CC*	26	7	Ref			18	4	Ref			8	3	Ref			26	8	Ref			18	5	Ref			8	3	Ref		
**rs7689099**	*CG*	367	89	0.71	0.33-1.54	0.39	237	54	0.85	0.31-2.36	0.76	129	35	0.49	0.15-1.61	0.24	367	136	1.04	0.51-2.13	0.91	237	80	1.03	0.42-2.55	0.94	129	56	1.04	0.33-3.34	0.94
	GG	1380	343	0.69	0.33-1.46	0.33	903	239	0.93	0.35-2.51	0.89	460	99	0.38	0.12-1.20	0.10	1380	492	0.94	0.47-1.89	0.86	903	333	1.07	0.44-2.59	0.88	460	152	0.72	0.23-2.26	0.57
	CG+GG	1747	432	0.69	0.33-1.46	0.33	1140	293	0.92	0.34-2.46	0.86	589	134	0.40	0.13-1.27	0.12	1747	628	0.96	0.48-1.93	0.91	1140	413	1.06	0.44-2.57	0.89	589	208	0.79	0.25-2.47	0.68
	CC+CG	393	96	Ref			255	58	Ref			137	38	Ref			393	144	Ref			255	85	Ref			137	59	Ref		
	GG	1380	343	0.94	0.75-1.18	0.60	903	239	1.07	0.80-1.42	0.66	460	99	0.73	0.50-1.06	0.10	1380	492	0.90	0.75-1.09	0.27	903	333	1.02	0.81-1.30	0.84	460	152	0.71	0.52-0.96	**0.02***
POLQ	*GG*	670	160	Ref			430	119	Ref			237	41	Ref			670	248	Ref			430	167	Ref			237	80	Ref		
**rs3218649**	*GC*	843	205	1.01	0.82-1.25	0.90	565	130	0.82	0.64-1.05	0.11	265	71	1.57	1.07-2.31	**0.02**	843	290	0.93	0.78-1.10	0.38	565	188	0.85	0.69-1.05	0.13	265	98	1.09	0.81-1.46	0.58
	CC	267	72	1.07	0.81-1.42	0.62	162	45	0.91	0.65-1.28	0.59	103	26	1.52	0.93-2.49	0.09	267	99	0.98	0.78-1.24	0.88	162	60	0.88	0.65-1.18	0.39	103	37	1.18	0.80-1.74	0.41
	GC+CC	1110	277	1.03	0.85-1.25	0.78	727	175	0.84	0.66-1.06	0.13	368	97	1.56	1.08-2.25	**0.02**	1110	839	0.94	0.80-1.10	0.44	727	248	0.86	0.70-1.04	0.12	368	135	1.11	0.84-1.46	0.46
	GG+GC	1513	365	Ref			995	249	Ref			502	112	Ref			1513	538	Ref			995	355	Ref			502	178	Ref		
	CC	267	72	1.07	0.83-1.37	0.62	162	45	1.02	0.74-1.40	0.91	103	26	1.18	0.77-1.80	0.46	267	99	1.03	0.83-1.27	0.82	162	60	0.97	0.74-1.27	0.80	103	37	1.11	0.78-1.59	0.55
REV1	*CC*	543	134	Ref			341	89	Ref			197	45	Ref			543	183	Ref			341	122	Ref			197	60	Ref		
**rs3087386**	*CT*	872	219	1.04	0.84-1.29	0.71	579	152	1.05	0.81-1.36	0.73	286	64	0.97	0.67-1.43	0.89	872	329	1.17	0.97-1.40	0.10	579	212	1.05	0.84-1.31	0.68	286	114	1.41	1.03-1.93	**0.03**
	TT	384	91	1.03	0.79-1.34	0.83	252	59	0.96	0.69-1.33	0.80	126	30	1.14	0.72-1.81	0.58	384	133	1.09	0.87-1.36	0.45	252	88	1.03	0.78-1.35	0.85	126	42	1.19	0.80-1.77	0.38

	CT+TT	1256	310	1.04	0.85-1.27	0.71	831	211	1.02	0.80-1.31	0.86	412	94	1.02	0.72-1.46	0.90	1256	462	1.14	0.96-1.36	0.12	831	300	1.04	0.85-1.29	0.70	412	156	1.35	1.00-1.81	**0.05**
	CC+CT	1415	353	Ref			920	241	Ref			483	109	Ref			1415	512	Ref			920	334	Ref			483	174	Ref		
	TT	384	91	1.01	0.80-1.27	0.96	252	59	0.93	0.70-1.24	0.63	126	30	1.16	0.78-1.74	0.47	384	133	0.99	0.82-1.20	0.93	252	88	1.00	0.79-1.26	0.97	126	42	0.96	0.69-1.35	0.83
**Austria**																															
EME1	*TT*	564	119	Ref			348	75	Ref			206	42	Ref			564	164	Ref			348	99	Ref			206	63	Ref		
**rs12450550**	*TC*	325	66	0.95	0.70-1.28	0.72	196	43	0.99	0.68-1.44	0.97	123	23	0.92	0.55-1.53	0.75	325	86	0.88	0.68-1.14	0.34	196	54	0.92	0.66-1.28	0.61	123	32	0.86	0.56-1.31	0.47
	CC	61	6	0.43	0.19-0.97	**0.04**	38	3	0.32	0.10-1.02	**0.05**	20	3	0.76	0.23-2.44	0.64	61	9	0.47	0.24-0.91	**0.03**	38	6	0.49	0.22-1.12	0.09	20	3	0.49	0.15-1.56	0.23
	TC+CC	386	72	0.86	0.64-1.15	0.31	234	46	0.87	0.61-1.26	0.47	143	26	0.90	0.55-1.47	0.67	386	95	0.81	0.63-1.05	0.10	234	60	0.85	0.61-1.16	0.30	143	35	0.81	0.53-1.22	0.30
	TT+TC	889	185	Ref			544	118	Ref			329	65	Ref			889	250	Ref			544	153	Ref			329	95	Ref		
	CC	61	6	0.44	0.19-0.99	**0.05**	38	3	0.32	0.10-1.02	**0.05**	20	3	0.78	0.24-2.47	0.67	61	9	0.49	0.25-0.95	**0.03**	38	6	0.51	0.22-1.14	0.10	20	3	0.52	0.17-1.64	0.26
FANCI	*CC*	333	74	Ref			211	48	Ref			118	26	Ref			333	106	Ref			211	66	Ref			118	40	Ref		
**rs2283432**	*CG*	462	87	0.83	0.61-1.13	0.23	284	57	0.86	0.59-1.26	0.44	165	28	0.75	0.44-1.29	0.30	462	115	0.74	0.57-0.96	**0.02***	284	73	0.78	0.56-1.09	0.14	165	40	0.66	0.43-1.03	0.07
	GG	155	30	0.86	0.57-1.32	0.50	87	16	0.81	0.46-1.42	0.45	66	14	0.96	0.50-1.84	0.90	155	38	0.75	0.51-1.08	0.12	87	20	0.71	0.43-1.17	0.18	66	18	0.78	0.45-1.37	0.39
	CG+GG	617	117	0.84	0.63-1.12	0.23	371	73	0.85	0.59-1.22	0.37	231	42	0.81	0.50-1.32	0.40	617	153	0.74	0.58-0.95	**0.02**	371	93	0.76	0.56-1.05	0.09	231	58	0.70	0.47-1.04	0.08
	CC+CG	795	161	Ref			495	105	Ref			283	54	Ref			795	221	Ref			495	139	Ref			283	80	Ref		
	GG	155	30	0.96	0.65-1.42	0.85	87	16	0.88	0.52-1.49	0.63	66	14	1.13	0.63-2.03	0.69	155	38	0.88	0.63-1.25	0.48	87	20	0.82	0.51-1.31	0.41	66	18	0.98	0.59-1.63	0.93
REV1	*CC*	301	50	Ref			191	30	Ref			102	20	Ref			301	71	Ref			191	42	Ref			102	29	Ref		
**rs3087386**	*CT*	481	106	1.38	0.99-1.93	0.06	298	67	1.55	1.01-2.39	**0.05**	175	38	1.06	0.61-1.81	0.85	481	139	1.28	0.97-1.71	0.09	298	85	1.40	0.97-2.03	0.07	175	53	1.04	0.66-1.64	0.86
	TT	168	35	1.31	0.85-2.02	0.22	93	24	1.81	1.06-3.10	**0.03**	72	10	0.67	0.32-1.44	0.31	168	49	1.30	0.90-1.87	0.16	93	32	1.73	1.09-2.74	**0.02**	72	16	0.74	0.40-1.36	0.33
	CT+TT	649	141	1.36	0.99-1.88	0.06	391	91	1.61	1.07-2.43	**0.02**	247	48	0.94	0.56-1.59	0.83	649	188	1.29	0.98-1.69	0.07	391	117	1.48	1.04-2.10	**0.03***	247	69	0.95	0.62-1.47	0.82
	CC+CT	782	156	Ref			489	97	Ref			277	58	Ref			782	210	Ref			489	127	Ref			277	82	Ref		
	TT	168	35	1.07	0.74-1.54	0.73	93	24	1.37	0.87-2.13	0.17	72	10	0.65	0.33-1.27	0.21	168	49	1.11	0.81-1.51	0.52	93	32	1.40	0.95-2.06	0.09	72	16	0.72	0.42-1.24	0.24
REV3L	*AA*	699	152	Ref			424	96	Ref			260	55	Ref			699	205	Ref			424	128	Ref			260	76	Ref		
**rs3204953**	*AG*	226	33	0.70	0.48-1.02	0.06	145	22	0.68	0.43-1.08	0.10	77	10	0.66	0.34-1.30	0.23	226	46	0.70	0.51-0.97	**0.03***	145	28	0.62	0.41-0.94	**0.02**	77	17	0.81	0.48-1.37	0.43
	GG	25	6	1.16	0.51-2.63	0.72	13	3	1.15	0.36-3.61	0.82	12	3	1.17	0.37-3.73	0.79	25	8	1.18	0.58-2.39	0.65	13	3	0.81	0.26-2.54	0.72	12	5	1.59	0.65-3.94	0.31
	AG+GG	251	39	0.75	0.52-1.06	0.10	158	25	0.72	0.46-1.11	0.14	89	13	0.74	0.40-1.35	0.32	251	54	0.75	0.55-1.01	0.06	158	31	0.64	0.43-0.94	**0.02***	89	22	0.91	0.57-1.47	0.70
	AA+AG	925	185	Ref			569	118	Ref			337	65	Ref			925	251	Ref			569	156	Ref			337	93	Ref		
	GG	25	6	1.25	0.56-2.83	0.59	13	3	1.25	0.40-3.94	0.70	12	3	1.26	0.40-4.01	0.70	25	8	1.27	0.63-2.57	0.50	13	3	0.90	0.29-2.82	0.85	12	5	1.67	0.68-4.10	0.27

CRC, colorectal cancer; HR, hazard ratio; CI, confidence interval.
Significant results in bold. Results that passed the Benjamini-Hochberg FDR test for multiple comparisons are marked with an asterisk.
[a] Numbers may not add up to 100% of available subjects because of genotyping failure. All samples that did not give a reliable result in the first round of genotyping were resubmitted to up to two additional rounds of genotyping. Data points that were still not filled after this procedure had been left blank.

Supplementary Table S2: SNPs associated with OS and EFS of patients with no received treatment in the pooled population and in groups stratified for tumor sites (Cox regression for adjusted estimates).

		Overall survival															*Event free survival*														
		All CRC patients					*Colon cancer patients*					*Rectal cancer patients*					*All CRC patients*					*Colon cancer patients*					*Rectal cancer patients*				
Gene SNP	Genotype	N[a]	Events	HR	95% CI	*p*-Value	N[a]	Events	HR	95% CI	*p*-Value	N[a]	Events	HR	95% CI	*p*-Value	N[a]	Events	HR	95% CI	*p*-Value	N[a]	Events	HR	95% CI	*p*-Value	N[a]	Events	HR	95% CI	*p*-Value
**Czech Republic**																															
MUS81	*CC*	82	24	Ref			52	18	Ref			29	6	Ref			82	33	Ref			52	24	Ref			29	9	Ref		
**rs545500**	*CG*	328	69	0.59	0.37-0.94	**0.03**	198	50	0.65	0.38-1.11	0.11	127	18	0.48	0.19-1.21	0.12	328	95	0.60	0.41-.090	**0.01***	198	64	0.63	0.39-1.00	**0.05**	127	30	0.58	0.27-1.21	0.15
	GG	363	99	0.80	0.52-1.26	0.34	237	59	0.63	0.37-1.06	0.08	124	39	1.35	0.57-3.19	0.49	363	124	0.76	0.52-1.12	0.17	237	70	0.58	0.36-0.92	**0.02**	124	53	1.31	0.65-2.65	0.46
	CG+GG	691	168	0.69	0.45-1.06	0.09	435	109	0.63	0.38-1.04	0.07	251	57	0.87	0.37-2.01	0.74	691	219	0.68	0.47-0.98	**0.04**	435	134	0.60	0.39-0.92	**0.02**	251	83	0.90	0.45-1.80	0.77
	CC+CG	410	93	Ref			250	68	Ref			156	24	Ref			410	128	Ref			250	88	Ref			156	39	Ref		
	GG	363	99	1.24	0.94-1.65	0.13	237	59	0.91	0.64-1.29	0.61	124	39	2.38	1.43-3.96	**0.0009***	363	124	1.14	0.89-1.46	0.30	237	70	0.83	0.61-1.14	0.26	124	53	2.06	1.36-3.11	**0.0007***
POLQ	*CC*	348	80	Ref			219	58	Ref			129	22	Ref			348	115	Ref			219	75	Ref			129	40	Ref		
**rs1381057**	*CT*	339	90	1.18	0.87-1.59	0.29	214	55	0.93	0.64-1.34	0.68	120	34	1.87	1.09-3.20	**0.02***	339	113	1.03	0.80-1.34	0.81	214	69	0.89	0.64-1.24	0.49	120	43	1.35	0.88-2.09	0.17
	TT	95	25	1.12	0.71-1.75	0.63	56	14	0.81	0.45-1.46	0.48	38	10	1.92	0.91-4.06	0.09	95	30	0.95	0.64-1.42	0.80	56	16	0.73	0.42-1.25	0.25	38	13	1.43	0.76-2.67	0.26
	CT+TT	434	115	1.16	0.87-1.55	0.30	270	69	0.90	0.63-1.27	0.55	158	44	1.88	1.13-3.14	**0.02***	434	143	1.01	0.79-1.30	0.92	270	85	0.85	0.63-1.16	0.32	158	56	1.37	0.91-2.05	0.13
	CC+CT	687	170	Ref			433	113	Ref			249	56	Ref			687	228	Ref			433	144	Ref			249	83	Ref		
	TT	95	25	1.03	0.68-1.57	0.88	56	14	0.85	0.49-1.48	0.56	38	10	1.39	0.71-2.72	0.34	95	30	0.94	0.64-1.38	0.75	56	16	0.77	0.46-1.30	0.33	38	13	1.23	0.68-2.20	0.49
POLQ	*GG*	301	69	Ref			189	52	Ref			112	17	Ref			301	101	Ref			189	67	Ref			112	34	Ref		
**rs3218649**	*GC*	360	91	1.64	0.85-1.59	0.34	229	55	0.89	0.61-1.30	0.54	126	35	2.03	1.14-3.63	**0.02***	360	112	0.98	0.75-1.28	0.88	229	68	0.85	0.60-1.19	0.33	126	43	1.27	0.81-2.00	0.29
	CC	110	32	1.30	0.86-1.98	0.22	65	19	0.97	0.58-1.65	0.92	44	12	2.25	1.07-4.71	**0.03**	110	41	1.18	0.82-1.70	0.37	65	23	0.93	0.58-1.49	0.75	44	17	1.76	0.98-3.15	0.06
	GC+CC	470	123	1.20	0.89-1.61	0.23	294	74	0.91	0.64-1.29	0.59	170	47	2.09	1.20-3.64	**0.009***	470	153	1.02	0.80-1.32	0.86	294	91	0.86	0.63-1.18	0.36	170	60	1.38	0.90-2.10	0.14
	GG+GC	661	160	Ref			418	107	Ref			238	52	Ref			661	213	Ref			418	135	Ref			238	77	Ref		
	CC	110	32	1.20	0.82-1.76	0.34	65	19	1.05	0.64-1.71	0.85	44	12	1.50	0.80-2.82	0.20	110	41	1.20	0.86-1.68	0.28	65	23	1.03	0.66-1.60	0.91	44	17	1.54	0.91-2.61	0.11
REV1	*AA*	557	135	Ref			358	92	Ref			195	41	Ref			557	185	Ref			358	116	Ref			195	67	Ref		
**rs3087399**	*AG*	210	54	1.05	0.76-1.44	0.77	125	33	1.05	0.71-1.57	0.80	84	21	1.09	0.64-1.84	0.76	210	64	0.86	0.64-1.14	0.28	125	41	0.97	0.68-1.38	0.86	84	23	0.70	0.43-1.12	0.14
	GG	19	6	1.60	0.71-3.63	0.26	13	4	1.37	0.51-3.74	0.53	6	2	2.55	0.61-10.62	0.20	19	9	1.76	0.90-3.44	0.10	13	5	1.26	0.51-3.08	0.61	6	4	4.89	1.74-13.74	**0.003***
	AG+GG	229	60	1.09	0.80-1.47	0.60	138	37	1.08	0.74-1.59	0.68	90	23	1.14	0.69-1.90	0.61	229	73	0.91	0.70-1.20	0.51	138	46	1.00	0.71-1.40	0.98	90	27	0.80	0.51-1.25	0.33
	AA+AG	767	189	Ref			483	125	Ref			279	62	Ref			767	249	Ref			483	157	Ref			279	90	Ref		
	GG	19	6	1.58	0.70-3.56	0.27	13	4	1.35	0.50-3.66	0.55	6	2	2.52	0.61-10.36	0.20	19	9	1.84	0.95-3.58	0.07	13	5	1.27	0.52-3.09	0.60	6	4	5.45	1.97-15.14	**0.001***
**Austria**																															
POLE	*AA*	306	49	Ref			210	38	Ref			86	10	Ref			306	56	Ref			210	42	Ref			86	13	Ref		
**rs5744934**	*AG*	68	14	1.28	0.71-2.33	0.41	48	11	1.26	0.64-2.46	0.50	19	2	0.87	0.19-3.96	0.85	68	16	1.29	0.74-2.25	0.37	48	12	1.24	0.65-2.36	0.51	19	3	1.01	0.29-3.56	0.98
	GG	3	2	4.01	0.97-16.49	**0.05**	2	2	5.59	1.34-23.24	**0.02**	1	0	NA			3	2	3.39	0.83-13.90	0.09	2	2	4.79	1.16-19.82	**0.03**	1	0	NA		
	AG+GG	71	16	1.40	0.80-2.47	0.24	50	13	1.43	0.76-2.68	0.27	20	2	0.82	0.18-3.75	0.80	71	18	1.39	0.82-2.36	0.23	50	14	1.39	0.76-2.54	0.29	20	3	0.96	0.27-3.37	0.95
	AA+AG	374	63	Ref			258	49	Ref			105	12	Ref			374	72	Ref			258	54	Ref			105	16	Ref		
	GG	3	2	3.83	0.94-15.64	0.06	2	2	5.40	1.31-22.29	**0.02**	1	0	NA			3	2	3.24	0.80-13.21	0.10	2	2	4.67	1.14-19.17	**0.03**	1	0	NA		

CRC, colorectal cancer; HR, hazard ratio; CI, confidence interval. Significant results in bold. Results that passed the Benjamini-Hochberg FDR test for multiple comparisons are marked with an asterisk. [a] Numbers may not add up to 100% of available subjects because of genotyping failure. All samples that did not give a reliable result in the first round of genotyping were resubmitted to up to two additional rounds of genotyping. Data points that were still not filled after this procedure had been left blank.

Supplementary Table S3: SNPs associated with OS and EFS of patients receiving 5-FU-based chemotherapy without oxaliplatin in the pooled population and in groups stratified for tumor sites (Cox regression for adjusted estimates).

Overall survival

		All CRC patients					*Colon cancer patients*					*Rectal cancer patients*				
**Gene SNP**	**Genotype**	**N[a]**	**Events**	**HR**	**95% CI**	***p*-Value**	**N[a]**	**Events**	**HR**	**95% CI**	***p*-Value**	**N[a]**	**Events**	**HR**	**95% CI**	***p*-Value**
**Czech Republic**																
FAAP24	*TT*	342	60	Ref			220	39	Ref			120	20	Ref		
**rs3816032**	*TC*	126	27	1.21	0.77-1.90	0.42	93	19	1.10	0.64-1.91	0.73	33	8	1.48	0.65-3.36	0.35
	CC	12	5	2.14	0.86-5.35	0.10	10	4	1.70	0.61-4.77	0.31	2	1	11.07	1.35-90.62	**0.03**
	TC+CC	138	32	1.29	0.84-1.98	0.24	103	23	1.17	0.70-1.97	0.54	35	9	1.63	0.74-3.58	0.23
	TT+TC	468	87	Ref			313	58	Ref			153	28	Ref		
	CC	12	5	1.97	0.80-4.85	0.14	10	4	1.61	0.58-4.45	0.36	2	1	6.43	0.84-49.18	0.07
RAD51D	*CC*	342	66	Ref			225	45	Ref			116	21	Ref		
**rs4796033**	*CT*	129	24	1.04	0.65-1.66	0.88	91	17	1.07	0.61-1.87	0.81	37	6	0.82	0.33-2.03	0.66
	TT	18	3	0.79	0.25-2.53	0.70	13	1	0.33	0.05-2.36	0.27	5	2	2.66	0.62-11.38	0.19
	CT+TT	147	27	1.00	0.64-1.57	0.99	104	18	0.95	0.55-1.64	0.85	42	8	0.99	0.44-2.25	0.99
	CC+CT	471	90	Ref			316	62	Ref			153	27	Ref		
	TT	18	3	0.78	0.25-2.47	0.67	13	1	0.32	0.04-2.28	0.25	5	2	2.74	0.65-11.55	0.17
REV1	*AA*	334	65	Ref			227	43	Ref			105	21	Ref		
**rs3087399**	*AG*	142	27	1.02	0.65-1.59	0.95	95	20	1.12	0.66-1.90	0.68	47	7	0.84	0.36-1.97	0.68
	GG	6	2	2.16	0.53-8.82	0.28	3	1	2.44	0.34-17.77	0.38	3	1	2.14	0.29-16.00	0.46
	AG+GG	148	29	1.06	0.68-1.63	0.81	98	21	1.15	0.68-1.94	0.60	50	8	0.91	0.40-.2.04	0.81
	AA+AG	476	92	Ref			322	63	Ref			152	28	Ref		
	GG	6	2	2.16	0.53-8.79	0.28	3	1	2.44	0.34-17.58	0.38	3	1	2.21	0.30-16.31	0.44
**Austria**																
POLN	*AA*	3	1	Ref			1	1	Ref			2	0	Ref		
**rs9328764**	*AG*	76	8	0.36	0.05-2.89	0.34	35	4	0.14	0.02-1.24	0.08	40	4	NA		
	GG	168	27	0.58	0.08-4.23	0.59	94	14	0.18	0.02-1.36	0.10	70	13	NA		
	AG+GG	244	35	0.51	0.07-3.69	0.50	129	18	0.17	0.02-1.25	0.08	110	17	NA		
	AA+AG	79	9	Ref			36	5	Ref			42	4	Ref		
	GG	168	27	1.46	0.69-3.11	0.32	94	14	1.14	0.41-3.17	0.80	70	13	1.95	0.64-5.99	0.24

Event free survival

		All CRC patients					*Colon cancer patients*					*Rectal cancer patients*				
**Gene SNP**	**Genotype**	**N[a]**	**Events**	**HR**	**95% CI**	***p*-Value**	**N[a]**	**Events**	**HR**	**95% CI**	***p*-Value**	**N[a]**	**Events**	**HR**	**95% CI**	***p*-Value**
**Czech Republic**																
FAAP24	*TT*	342	110	Ref			220	70	Ref			120	38	Ref		
**rs3816032**	*TC*	126	37	0.89	0.62-1.30	0.55	93	25	0.79	0.50-1.25	0.32	33	12	1.18	0.61-2.25	0.63
	CC	12	6	1.27	0.56-2.90	0.56	10	4	0.85	0.31-2.34	0.76	2	2	7.43	1.73-32.02	**0.007***
	TC+CC	138	43	0.93	0.66-1.33	0.69	103	29	0.80	0.52-1.24	0.32	35	14	1.34	0.72-2.47	0.36
	TT+TC	468	147	Ref			313	95	Ref			153	50	Ref		
	CC	12	6	1.32	0.58-2.99	0.50	10	4	0.94	0.34-2.55	0.90	2	2	7.27	1.72-30.69	**0.007***
RAD51D	*CC*	342	111	Ref			225	70	Ref			116	40	Ref		
**rs4796033**	*CT*	129	35	0.85	0.58-1.24	0.40	91	25	0.98	0.62-1.55	0.93	37	9	0.59	0.29-1.21	0.15
	TT	18	9	1.49	0.75-2.93	0.25	13	5	1.02	0.41-2.52	0.97	5	4	3.98	1.40-11.33	**0.01***
	CT+TT	147	44	0.93	0.66-1.32	0.68	104	30	0.98	0.64-1.51	0.94	42	13	0.80	0.43-1.49	0.48
	CC+CT	471	146	Ref			316	95	Ref			153	49	Ref		
	TT	18	9	1.54	0.78-3.01	0.21	13	5	1.01	0.41-2.49	0.98	5	4	4.53	1.61-12.78	**0.004***
REV1	*AA*	334	111	Ref			227	70	Ref			105	39	Ref		
**rs3087399**	*AG*	142	42	0.89	0.62-1.27	0.52	95	29	0.96	0.62-1.48	0.86	47	13	0.80	0.43-1.49	0.48
	GG	6	3	1.76	0.56-5.55	0.33	3	2	4.10	1.00-16.91	**0.05**	3	1	0.87	0.12-6.34	0.89
	AG+GG	148	45	0.92	0.65-1.30	0.64	98	31	1.01	0.66-1.54	0.96	50	14	0.80	0.43-1.47	0.47
	AA+AG	476	153	Ref			322	99	Ref			152	52	Ref		
	GG	6	3	1.83	0.58-5.74	0.30	3	2	4.00	0.98-16.33	**0.05**	3	1	0.93	0.13-6.70	0.94
**Austria**																
POLN	*AA*	3	1	Ref			1	1	Ref			2	0	Ref		
**rs9328764**	*AG*	76	17	0.61	0.08-4.58	0.63	35	7	0.03	0.002-0.52	**0.02**	40	10	NA		
	GG	168	37	0.61	0.08-4.48	0.63	94	19	0.08	0.01-0.64	**0.02**	70	18	NA		
	AG+GG	244	54	0.61	0.09-4.42	0.63	129	26	0.06	0.01-0.52	**0.01**	110	28	NA		
	AA+AG	79	18	Ref			36	8	Ref			42	10	Ref		
	GG	168	37	0.95	0.54-1.67	0.86	94	19	0.90	0.39-2.05	0.80	70	18	1.05	0.49-2.28	0.90

CRC, colorectal cancer; HR, hazard ratio; CI, confidence interval.

Significant results in bold. Results that passed the Benjamini-Hochberg FDR test for multiple comparisons are marked with an asterisk.

[a] Numbers may not add up to 100% of available subjects because of genotyping failure. All samples that did not give a reliable result in the first round of genotyping were resubmitted to up to two additional rounds of genotyping. Data points that were still not filled after this procedure had been left blank.

Supplementary Table S4: SNPs associated with OS and EFS of patients receiving 5-FU in combination with oxaliplatin in the pooled population and in groups stratified for tumor sites (Cox regression for adjusted estimates).

		Overall survival															*Event free survival*														
		All CRC patients					*Colon cancer patients*					*Rectal cancer patients*					*All CRC patients*					*Colon cancer patients*					*Rectal cancer patients*				
**Gene SNP**	**Genotype**	**N[a]**	**Events**	**HR**	**95% CI**	*p*-Value	**N[a]**	**Events**	**HR**	**95% CI**	*p*-Value	**N[a]**	**Events**	**HR**	**95% CI**	*p*-Value	**N[a]**	**Events**	**HR**	**95% CI**	*p*-Value	**N[a]**	**Events**	**HR**	**95% CI**	*p*-Value	**N[a]**	**Events**	**HR**	**95% CI**	*p*-Value
																**Czech Republic**															
FAAP24	*TT*	232	96	Ref			175	72	Ref			56	24	Ref			232	144	Ref			175	108	Ref			56	36	Ref		
**rs3816032**	*TC*	65	18	0.57	0.34-0.94	**0.03**	44	11	0.51	0.27-0.96	**0.04**	20	7	0.74	0.32-1.72	0.48	65	36	0.80	0.56-1.16	0.24	44	23	0.75	0.48-1.18	0.21	20	13	1.01	0.53-1.90	0.98
	CC	2	1	1.00	0.14-7.17	1.00	2	1	1.03	0.14-7.42	0.98	0	0	NA			2	2	3.89	0.96-15.84	0.06	2	2	3.93	0.96-16.10	0.06	0	0	NA		
	TC+CC	67	19	0.58	0.35-0.95	**0.03**	46	12	0.53	0.29-0.98	**0.04**	20	7	0.74	0.32-1.72	0.48	67	38	0.84	0.59-1.20	0.34	46	25	0.80	0.52-1.24	0.32	20	13	1.01	0.53-1.90	0.98
	TT+TC	297	114	Ref			219	83	Ref			76	31	Ref			297	180	Ref			219	131	Ref			76	49	Ref		
	CC	2	1	1.13	0.16-8.06	0.91	2	1	1.17	0.16-8.37	0.88	0	0	NA			2	2	3.93	0.97-15.93	0.06	2	2	4.01	0.98-16.31	**0.05**	0	0	NA		
MUS81	*CC*	21	9	Ref			11	6	Ref			9	3	Ref			21	10	Ref			11	7	Ref			9	3	Ref		
**rs545500**	*CG*	140	50	0.82	0.40-1.66	0.57	103	35	0.59	0.25-1.39	0.22	36	15	1.58	0.45-5.47	0.47	140	86	1.43	0.74-2.75	0.29	103	61	0.90	0.41-1.96	0.78	36	25	3.28	0.98-10.98	**0.05**
	GG	135	53	0.97	0.48-1.97	0.93	102	39	0.69	0.29-1.64	0.40	33	14	1.94	0.55-6.81	0.30	135	83	1.51	0.78-2.91	0.22	102	61	0.89	0.41-1.94	0.77	33	22	4.21	1.20-14.75	**0.02**
	CG+GG	275	103	0.89	0.45-1.75	0.73	205	74	0.63	0.28-1.46	0.28	69	29	1.76	0.53-5.79	0.36	275	169	1.48	0.78-2.79	0.23	205	122	0.89	0.42-1.92	0.77	69	47	3.73	1.15-12.14	**0.03**
	CC+CG	161	59	Ref			114	41	Ref			45	18	Ref			161	96	Ref			114	68	Ref			45	28	Ref		
	GG	135	53	1.15	0.79-1.67	0.46	102	39	1.12	0.72-1.73	0.63	33	14	1.27	0.63-2.56	0.50	135	83	1.14	0.85-1.53	0.38	102	61	1.03	0.73-1.45	0.88	33	22	1.59	0.90-2.80	0.11
POLE	*AA*	212	78	Ref			158	56	Ref			52	22	Ref			212	129	Ref			158	92	Ref			52	37	Ref		
**rs5744934**	*AG*	83	37	1.21	0.82-1.79	0.34	57	27	1.29	0.82-2.05	0.27	26	10	0.96	0.46-2.03	0.92	83	49	0.94	0.68-1.31	0.71	57	36	1.04	0.71-1.53	0.85	26	13	0.66	0.35-1.25	0.20
	GG	4	1	0.61	0.08-4.37	0.62	4	1	0.64	0.09-4.63	0.66	0	0	NA			4	4	3.07	1.12-8.42	**0.03**	4	4	3.21	1.16-8.90	**0.03**	0	0	NA		
	AG+GG	87	38	1.18	0.80-1.73	0.41	61	28	1.25	0.79-1.96	0.34	26	10	0.96	0.46-2.03	0.92	87	53	0.99	0.72-1.36	0.95	61	40	1.11	0.77-1.62	0.57	26	13	0.66	0.35-1.25	0.20
	AA+AG	295	115	Ref			215	83	Ref			78	32	Ref			295	178	Ref			215	128	Ref			78	50	Ref		
	GG	4	1	0.56	0.08-4.02	0.57	4	1	0.58	0.08-4.18	0.59	0	0	NA			4	4	3.24	1.19-8.82	**0.02**	4	4	3.36	1.22-9.25	**0.02***	0	0	NA		
POLQ	*CC*	132	50	Ref			101	39	Ref			30	11	Ref			132	82	Ref			101	62	Ref			30	20	Ref		
**rs1381057**	*CT*	129	46	0.94	0.63-1.40	0.75	98	35	0.88	0.56-1.39	0.58	30	11	1.22	0.53-2.82	0.65	129	71	0.94	0.68-1.29	0.69	98	54	0.91	0.63-1.31	0.62	30	17	1.01	0.53-1.94	0.97
	TT	29	15	1.43	0.80-2.55	0.22	17	9	1.50	0.73-3.10	0.27	12	6	1.32	0.49-3.57	0.59	29	22	1.41	0.88-2.26	0.15	17	14	1.98	1.11-3.54	**0.02**	12	8	0.82	0.36-1.87	0.64
	CT+TT	158	61	1.02	0.70-1.49	0.90	115	44	0.96	0.62-1.48	0.85	42	17	1.23	0.58-2.64	0.59	158	93	1.02	0.76-1.37	0.91	115	68	1.02	0.73-1.44	0.90	42	25	0.94	0.52-1.70	0.85
	CC+CT	261	96	Ref			199	74	Ref			60	22	Ref			261	153	Ref			199	116	Ref			60	37	Ref		
	TT	29	15	1.48	0.56-2.54	0.16	17	9	1.63	0.81-3.25	0.17	12	6	1.14	0.46-2.82	0.78	29	22	1.46	0.93-2.28	0.10	17	14	2.06	1.18-3.59	**0.01***	12	8	0.83	0.38-1.78	0.63
POLQ	*AA*	213	92	Ref			155	69	Ref			57	23	Ref			213	134	Ref			155	97	Ref			57	37	Ref		
**rs3218651**	*AG*	80	22	0.59	0.37-0.93	**0.02**	61	16	0.51	0.29-0.87	**0.01***	18	6	0.98	0.40-2.41	0.96	80	43	0.76	0.54-1.07	0.12	61	33	0.74	0.50-1.10	0.14	18	10	0.83	0.41-1.67	0.60
	GG	7	1	0.23	0.03-1.64	0.14	6	0	NA			1	1	2.21	0.30-16.64	0.44	7	5	0.74	0.30-1.81	0.51	6	4	0.71	0.26-1.92	0.49	1	1	0.90	0.12-6.59	0.91
	AG+GG	87	23	0.55	0.35-0.86	**0.01***	67	16	0.45	0.26-0.77	**0.004***	19	7	1.07	0.46-2.48	0.89	87	48	0.76	0.54-1.05	0.10	67	37	0.73	0.50-1.07	0.11	19	11	0.84	0.43-1.64	0.60
	AA+AG	293	114	Ref			216	85	Ref			75	29	Ref			293	177	Ref			216	130	Ref			75	47	Ref		
	GG	7	1	0.26	0.04-1.86	0.18	6	0	NA			1	1	2.25	0.30-16.75	0.43	7	5	0.79	0.32-1.92	0.60	6	4	0.76	0.28-2.06	0.59	1	1	0.91	0.13-6.67	0.93
REV3L	*GG*	214	82	Ref			158	61	Ref			54	21	Ref			214	127	Ref			158	92	Ref			54	35	Ref		
**rs3204953**	*GA*	76	33	1.13	0.75-1.69	0.55	54	22	0.96	0.59-1.56	0.86	22	11	1.81	0.87-2.77	0.11	76	52	1.27	0.92-1.76	0.14	54	37	1.23	0.34-1.80	0.29	22	15	1.35	0.74-2.48	0.33
	AA	9	1	0.26	0.04-1.86	0.18	7	0	NA			2	1	NA			9	4	0.76	0.28-2.04	0.58	7	3	0.62	0.20-1.94	0.41	2	1	3.82	0.49-30.05	0.20
	GA+AA	85	34	1.03	0.69-1.53	0.89	61	22	0.82	0.51-1.34	0.43	24	12	1.96	0.96-4.00	0.07	85	56	1.21	0.89-1.66	0.23	61	40	1.14	0.79-1.66	0.48	24	16	1.40	0.77-2.54	0.26
	GG+GA	290	115	Ref			212	83	Ref			76	32	Ref			290	179	Ref			212	129	Ref			76	50	Ref		
	AA	9	1	0.25	0.04-1.79	0.17	7	0	NA			2	1	35.51	3.22-391.55	**0.004***	9	4	0.71	0.26-1.92	0.50	7	3	0.58	0.19-1.84	0.36	2	1	3.08	0.41-23.35	0.28
																**Austria**															
RAD51D	*CC*	178	51	Ref			112	30	Ref			65	21	Ref			178	76	Ref			112	47	Ref			65	29	Ref		
**rs4796033**	*CT*	68	19	0.92	0.54-1.55	0.75	46	13	0.99	0.52-1.90	0.97	21	6	0.84	0.34-2.09	0.71	68	31	1.02	0.67-1.55	0.93	46	20	1.03	0.61-1.74	0.91	21	11	1.04	0.52-2.09	0.90
	TT	6	2	1.51	0.37-6.20	0.57	5	1	0.98	0.13-	0.99	1	1	4.26	0.55-	0.16	6	2	0.98	0.24-	0.98	5	1	0.53	0.07-	0.53	1	1	9.39	1.13-	**0.04**

										7.20					32.77					3.99					3.84					78.00	
	CT+TT	74	21	0.95	0.57-1.58	0.85	51	14	0.99	0.52-1.86	0.97	22	7	0.95	0.40-2.24	0.91	74	33	1.02	0.68-1.53	0.94	51	21	0.99	0.59-1.65	0.96	22	12	1.13	0.58-2.21	0.73
	CC+CT	246	70	Ref			158	43	Ref			86	27	Ref			246	107	Ref			158	67	Ref			86	40	Ref		
	TT	6	2	1.55	0.38-6.30	0.54	5	1	0.98	0.14-7.15	0.99	1	1	4.28	0.57-32.30	0.16	6	2	0.96	0.24-3.90	0.96	5	1	0.53	0.07-3.80	0.53	1	1	10.86	1.34-88.28	**0.03**
REV3L	*GG*	186	57	Ref			120	35	Ref			64	22	Ref			186	88	Ref			120	55	Ref			64	33	Ref		
**rs3204953**	*GA*	62	13	0.71	0.39-1.30	0.27	41	7	0.60	0.27-1.36	0.22	21	6	0.87	0.35-2.14	0.76	62	19	0.62	0.38-1.02	0.06	41	11	0.55	0.29-1.05	0.07	21	8	0.73	0.34-1.59	0.43
	AA	4	2	2.30	0.56-9.44	0.25	2	2	53.33	8.70-327.02	**<.0001***	2	0	NA			4	2	1.19	0.29-4.81	0.81	2	2	6.24	1.45-26.83	**0.01***	2	0	NA		
	GA+AA	66	15	0.78	0.44-1.39	0.40	43	9	0.77	0.37-1.61	0.49	23	6	0.76	0.31-1.88	0.56	66	21	0.65	0.40-1.04	0.07	43	13	0.64	0.35-1.17	0.15	23	8	0.64	0.30-1.39	0.26
	GG+GA	248	70	Ref			161	42	Ref			85	28	Ref			248	107	Ref			161	66	Ref			85	41	Ref		
	AA	4	2	2.51	0.62-10.25	0.20	2	2	70.36	11.47-431.53	**<.0001***	2	0	NA			4	2	1.32	0.33-5.34	0.70	2	2	7.45	1.75-31.81	**0.007***	2	0	NA		

CRC, colorectal cancer; HR, hazard ratio; CI, confidence interval; NA, not applicable.

Significant results in bold. Results that passed the Benjamini-Hochberg FDR test for multiple comparisons are marked with an asterisk.

[a] Numbers may not add up to 100% of available subjects because of genotyping failure. All samples that did not give a reliable result in the first round of genotyping were resubmitted to up to two additional rounds of genotyping. Data points that were still not filled after this procedure had been left blank.

Add 2.4. Survival and Therapy

Group 1 (**Supplementary Table S2**)

Czech sample set. In the group of CRC patients receiving no treatment, carriers of the *CG* genotype in *MUS81* rs545500 were associated with increased EFS (*CG* vs. *CC*; HR 0.60; 95% CI 0.41-0.90; P = 0.01 and *CG*+*GG* vs. *CC*; HR 0.68; 95% CI 0.47-0.98; P = 0.04). The association remained significant by tumor sub-site. Although the decreased EFS (and OS as well) was observed in the recessive model for rectal cancer patients (*GG* vs. *CC*+*CG* OS: HR 2.38; 95% CI 1.43-3.96; P = 0.0009 and EFS: HR 2.06; 95% CI 1.36-3.11; P = 0.0007).

In rectal cancer patients, rs3218649 and rs1381057 in the *POLQ* gene were significantly associated with OS in the codominant and dominant models (rs3218649 *GC*+*CC* vs. *GG*; HR 2.09; 95% CI 1.20-3.64; P = 0.009; rs1381057 *CT*+*TT* vs. *CC*; HR 1.88; 95% CI 1.13-3.14; P = 0.02). Additionally, carriers of the variant *GG* genotype in *REV1* rs3087399 showed decreased EFS when compared with the most frequent *AA* genotype (HR 4.89; 95% CI 1.74-13.74; P = 0.003) or with A-allele carriers (HR 5.45; 95% CI 1.97-15.14; P = 0.001). However, in this case given the low numbers of patients with the variant genotype, the result should be considered with caution.

Austrian sample set. In colon cancer patients, carriers of the variant *GG* genotype in *POLE* rs5744934 were associated with decreased OS and EFS in both codominant and recessive models (*GG* vs. *AA*+*AG* OS: HR 5.40; 95% CI 1.31-22.29; P = 0.02 and EFS: HR 4.67; 95% CI 1.14-19.17; P = 0.03).

Group 2 (**Supplementary Table S3**)

Czech sample set. In patients undergoing 5-FU-based chemotherapy without oxaliplatin, there was no significant association with survival outcomes by genotype for all CRC patients. For colon cancer, only the homozygous variant genotype in *REV1* rs3087399 was marginally significantly associated with decreased OS (*GG* vs. *AA*; HR 4.10; 95% CI 1.00-16.91; P = 0.05). Two SNPs were associated with survival in rectal cancer patients. The *TT* variant genotype in *RAD51D* rs4796033 was associated with decreased OS in the codominant and recessive models (*TT* vs. *CC*; HR 3.98; 95% CI 1.40-11.33; P = 0.01; *TT* vs. *CC*+*CT*; HR 4.53; 95% CI 1.61-12.78; P = 0.004) while the variant *CC* genotype in *FAAP24* rs38116032 was associated with both OS and EFS in different genetic models (although with wide confidence intervals).

Austrian sample set. In colon cancer patients, the variant G-allele carriers in *POLN* rs9328764 were associated with increased EFS in codominant and dominant models (*GG* vs. *AA*; HR 0.08; 95% CI 0.01-0.64; P = 0.02 and *AG*+*GG* vs. *AA*; HR 0.06; 95% CI 0.01-0.52; P = 0.01).

Group 3 (**Supplementary Table S4**)

Czech sample set. Considering all CRC patients receiving 5-FU in combination with oxaliplatin, carriers of heterozygous genotypes in *FAAP24* rs3816032 and *POLQ* rs3218651 were associated with increased OS. In both cases the significance was reached in the codominant and dominant models, however, due to the low frequency of the homozygous variant genotypes in our patient group it was not possible to observe the same effect in the codominant model. Additionally, carriers of the variant *GG* genotype in *POLE* rs5744934 showed decreased EFS (*GG* vs. *AA*; HR 3.07; 95% CI 1.12-8.42; P = 0.03 and *GG* vs. *AA*+*AG*; HR 3.24; 95% CI 1.19-8.82; P = 0.02). The association of all three SNPs remained significant after stratification according to tumor localization. In colon cancer patients, two more SNPs were associated with decreased EFS (*CC* genotype in *FAAP24* rs3816032: *CC* vs. *TT*+*TC*; HR 4.01; 95% CI 0.98-16.31; P = 0.05 and *TT* genotype in *POLQ* rs1381057: *TT* vs. *CC*; HR 1.98; 95% CI 1.11-3.54; P = 0.02 and *TT* vs. *CC*+*CT*; HR 2.06; 95% CI 1.18-3.59; P = 0.01).

In rectal cancer patients two notable associations were detected: Carriers of the variant *AA* genotype in *REV3L* rs3204953 showed worse OS (*AA* vs. *GG*+*GA*; HR 35.51; 95% CI 3.22-391.55; P = 0.004) and

carriers of the variant allele G in *MUS81* rs545500 displayed decreased EFS (*GG* vs. *CC*; HR 4.21; 95% CI 1.20-14.75; P = 0.02 and *CG+GG* vs. *CC*; HR 3.73; 95% CI 1.15-12.14, P = 0.03).

Austrian sample set. In colon cancer patients, carriers of the variant AA genotype in *REV3L* rs3204953 were associated with decreased OS and EFS in codominant and recessive models (*AA* vs. *GG+GA* OS: HR 7.36; 95% CI 11.47-431.53; P < 0.0001 and EFS: HR 7.45; 95% CI 1.75-31.81; P = 0.007). In rectal cancer patients, rs4796033 in the *RAD51D* gene was significantly associated with EFS in the codominant and recessive models (*TT* vs. *CC*; HR 9.39; 95% CI 1.13-78.00; P = 0.04 and *TT* vs. *CC+CT*; HR 10.86; 95% CI 1.34-88.28; P = 0.03).
